# Spin-Vibronic Intersystem Crossing and Molecular Packing Effects in Heavy Atom Free Organic Phosphor

**DOI:** 10.1021/acs.jctc.3c01220

**Published:** 2024-01-25

**Authors:** Thomas Pope, Julien Eng, Andrew Monkman, Thomas J. Penfold

**Affiliations:** † Chemistry, School of Natural and Environmental Sciences, Newcastle University, Newcastle upon Tyne NE1 7RU, U.K.; ‡ Department of Physics, Durham University, South Road, Durham DH1 3LE, U.K.

## Abstract

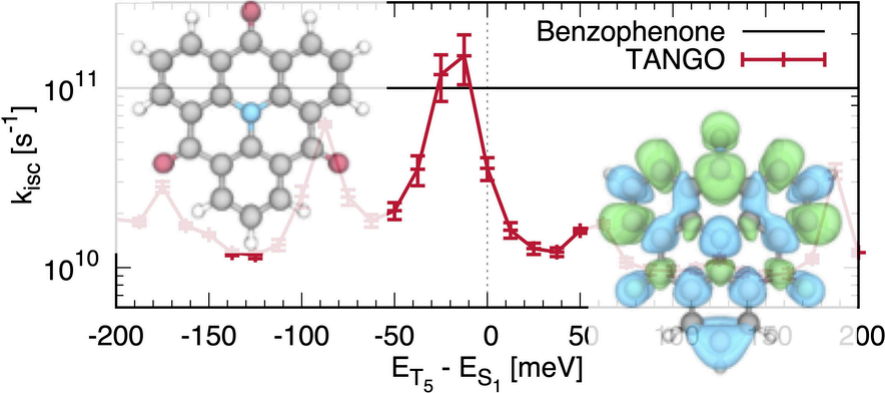

We present a detailed investigation into the excited state properties of a planar *D*_3*h*_ symmetric azatriangulenetrione, HTANGO, which has received significant interest due to its high solid-state phosphorescence quantum yield and therefore potential as an organic room temperature phosphorescent (ORTP) dye. Using a model linear vibronic coupling Hamiltonian in combination with quantum dynamics simulations, we observe that intersystem crossing (ISC) in HTANGO occurs with a rate of ∼10^10^ s^–1^, comparable to benzophenone, an archetypal molecule for fast ISC in heavy metal free molecules. Our simulations demonstrate that the mechanism for fast ISC is associated with the high density of excited triplet states which lie in close proximity to the lowest singlet states, offering multiple channels into the triplet manifold facilitating rapid population transfer. Finally, to rationalize the solid-state emission properties, we use quantum chemistry to investigate the excited state surfaces of the HTANGO dimer, highlighting the influence and importance of the rotational alignment between the two HTANGO molecules in the solid state and how this contributes to high phosphorescence quantum yield.

## Introduction

Purely organic molecules exhibiting a strong coupling between the singlet and triplet manifolds have attracted a significant research effort in both fundamental^1−7^ as well as applied research fields.^8−12^ Indeed, for the latter, organic molecules which display efficient population transfer between singlet and triplet states have the potential to be applied as key components in organic light emitting diodes operating via thermally activated delayed fluorescence^8,13−15^ or in photodynamic therapy,^16,17^ photocatalysts^18−20^ and molecular logic gates.^21^ Besides excited state switching between singlet and triplet states, there is also a surging interest in organic room temperature phosphorescent dyes as substitutes for conventional inorganic or metal–organic phosphors.^9,22−25^

In most organic molecules, the coupling between the singlet and triplet manifolds is weak, making intersystem crossing (ISC) and phosphorescence uncompetitive compared to spin-allowed transitions such as fluorescence and nonradiative decay.^26,27^ The simplest approach for enhancing spin state mixing is by increasing spin–orbit coupling (SOC) through the heavy-atom effect by incorporating atoms such as Br or I into the material.^28,29^ However, alternative approaches have sought to exploit the twist in the molecular structure^7,30−33^ and increase the density of low lying triplet excited states.^34−38^ Alternatively, as outlined by El-Sayed,^39,40^ SOC can be enhanced by tuning the character of the states involved. To achieve effective SOC, any change in spin must be accompanied by a corresponding change in orbital angular momentum, so that total angular momentum is conserved and such conditions are fulfilled by coupling *n*–π* and π–π* states, which can be realized using carbonyl (C=O) containing derivatives.

One of the most widely studied examples of ISC dynamics in heavy metal free organic molecules is benzophenone, which exhibits ISC in the picosecond regime.^42−47^ Excited state dynamics simulations of benzophenone performed by Favero et al.^48^ demonstrated that direct (i.e., *S*_1_ → *T*_1_) ISC accounted for the majority of the spin-flipping dynamics, with the remaining crossing to the *T*_2_ or higher triplet states, which subsequently undergo ultrafast nonradiative decay into the *T*_1_ state. Their simulations ascribed the fast ISC to two internal coordinates activated after *n*–π* excitation, namely, the C=O stretch and the torsion of phenyl rings. Further supporting this, Karak et al.^49^ recently demonstrated that quenching of the latter (i.e., the torsion of phenyl rings) using the fused analogue of benzophenone, fluorenone, reduced the rate of ISC by 3 orders of magnitude.

**Figure 1:**
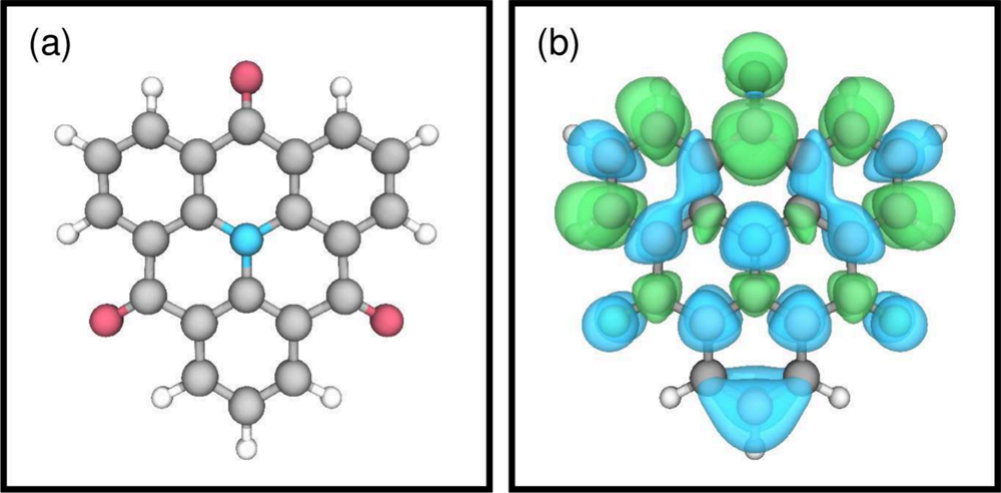
(a) Representation of the structure of the unsubstituted azatriangulenetriones, HTANGO.^41^ (b) Difference of electronic density associated with the S_0_ → *S*_1_ transition at the ground state geometry.

In 1971, Hellwinkel et al.^50^ synthesized a series of azatriangulenetriones, the archetypal of which is shown in Figure 1 and from now on will be referred to as HTANGO. The crystal structure was determined by Field and Venkataraman,^51^ and related complexes have recently been synthesized with the objective of achieving molecules which exhibit narrow emission for application in OLEDs.^52,53^ Hamzehpoor and Perepichka^41^ have studied the luminescent properties of HTANGO and related derivatives in solution and the solid state. Despite the rigidity of the structure, found to be detrimental for the ISC of benzophenone, the authors demonstrated that HTANGO exhibited a solid-state phosphorescence quantum yield of 42% at room temperature and is the most efficient phosphor composed of first- and second-row elements only. Consequently, toward achieving a detailed understanding of its photophysics, especially in the context of ISC and phosphorescence, in this work, we combine quantum chemistry and quantum dynamics to study the excited state dynamics of HTANGO. We show, employing a model linear vibronic coupling Hamiltonian and conducting quantum dynamics simulations, that HTANGO undergoes ISC with a rate of approximately 10^10^ s^–1^, comparable to benzophenone, driven by exploiting the high density of low lying triplet excited states. Additionally, we employ quantum chemistry to explore the excited state surfaces of HTANGO dimers and emphasize the significance of rotational alignment between HTANGO monomers to elucidate the solid-state emission properties. Overall, this study provides valuable insights into the excited state behavior of HTANGO leading to potential applications as an ORTP dye.

## Theory

### Quantum Chemistry

All density functional theory (DFT), time-dependent density functional theory (TD-DFT), and equation-of-motion coupled cluster (EOM-CCSD) theory calculations were performed with the ORCA 5 quantum chemistry package.^54−56^ All calculations employed a def2-TZVP basis set and, where applicable, the corresponding auxiliary /C and /J basis sets.^57,58^ A linear-response conductor-like polarizable continuum model with the properties of chloroform was used in all cases.^59^ DFT/TD-DFT simulations were performed within the approximation of the PBE0 exchange-correlation functional^60,61^ and a pairwise dispersion correction.^62,63^ All TD-DFT calculations were performed within the Tamm–Dancoff approximation.^64^

### Linear Vibronic Coupling Hamiltonian

The Linear Vibronic Coupling (LVC) Hamiltonian^65,66^ has previously been widely used to study the photophysics,^67−70^ including intersystem crossing^71−75^ of molecular systems. It comprises a base Hamiltonian and four additional terms defining four coupling mechanisms,
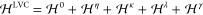
1The base Hamiltonian for the system is constructed from the normal modes *Q*_*k*_, their frequencies ω_*k*_, and the vertical excitation energies ϵ_*n*_.
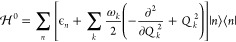
2The spin–orbit coupling (SOC) η^*mn*^ is calculated using quasi-degenerate perturbation theory^76^ and the gradient of the spin–orbit coupling with respect to the normal modes *∂η*^*mn*^/*∂Q*_*i*_ is estimated using a finite-difference method. [Note: Here, the molecule is displaced along the normal mode by increments of 0.1 dimensionless normal coordinates. The gradient is calculated with a linear fit.] With these components, the SOC Hamiltonian is given,
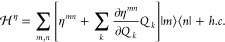
3Owing to the *D*_3*h*_ point group symmetry of the HTANGO molecule, three LVC terms are considered. For fully symmetric normal modes, intrastate coupling, κ_*n*_, is included for all states,
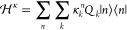
4Otherwise, when the direct product between the irreducible representations (irreps) of the electronic states, Γ_*m*/*n*_, and the normal modes, Γ_*Q*_*k*__, contains the fully symmetric irrep (Γ_*m*_ ⊗ Γ_*Q*_*k*__ ⊗ Γ_*n*_ ⊇ Γ_A_1_′_), interstate vibronic coupling, λ_*k*_^*mn*^, is induced,
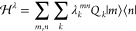
5with the exception of degenerate states of *E*′ or *E*″ symmetry. In this case, the pair of states, *n* and *n*′, is coupled by a degenerate pair of normal modes, *k* and *k*′, via Jahn–Teller (JT) coupling,
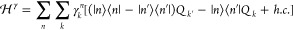
6

**Figure 2:**
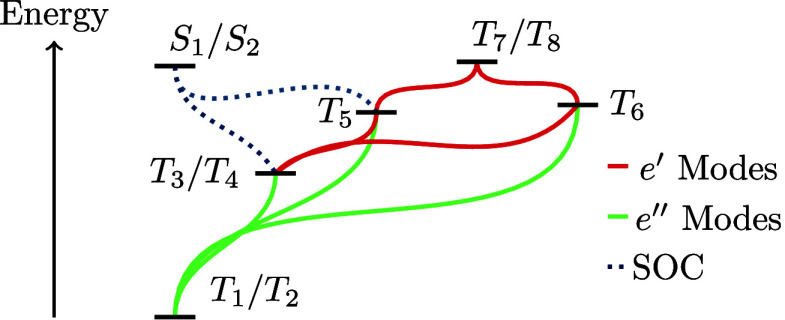
Schematic of the HTANGO monomer and the coupling pathways through spin–orbit coupling (blue-dashed) and LV coupling through the *e*′ (red) and *e*″ (green) modes. The states are vertically distributed following their energies.

We consider the lowest singlet states, the degenerate pair *S*_1_/*S*_2_, and the eight triplet states of similar or lower energy. Of the triplet states, there are three degenerate pairs, *T*_1_/*T*_2_, *T*_3_/*T*_4_, and *T*_7_/*T*_8_, and two nondegenerate states, *T*_5_ and *T*_6_. Figure 2 shows a schematic of the states and the coupling pathways available. The spin–orbit coupling induces transfer between the singlet states and *T*_3_/*T*_4_ and *T*_5_ states. The higher triplet states all couple through the *e*′ modes and the triplet pair *T*_1_/*T*_2_ couples to the higher triplet states through *e*″ modes. JT coupling occurs between degenerate pairs of states along *e*′ modes.

Intrastate coupling terms are derived from the Franck–Condon (FC) geometry using the method outlined by Eng et al.^77^ The JT terms were calculated in the same way, using the gradient of the adiabatic state at the FC geometry.

For interstate coupling, the approach was modified. In the standard approach, the interstate coupling between two states is given by the second derivatives along the normal modes for each state involved,
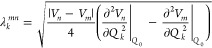
7However, this two-state approach assumes the change in gradient along a normal mode for a given state is caused by only one other state, which is not always the case, especially for molecules exhibiting a high density of states. For example in the present case of HTANGO, as shown in Figure 2, *T*_6_ (which has *A*_1_′ symmetry) is expected to vibronically couple to two degenerate pairs, *T*_3_/*T*_4_ and *T*_7_/*T*_8_ (both of *E*′ symmetry), via *e*′ modes. This vibronic coupling via a given mode leads to a repulsion between the states along that mode, which increases the gradiant for states coupled to lower-energy states, and decreases the gradient for states coupled to higher-energy states. In the above case, we would expect the gradient of the *T*_5_ energy to be increased due to the *T*_3_/*T*_4_ pair and reduced due to the *T*_7_/*T*_8_ and, since these behaviors will both be observed, the change in gradient for this state along any *e*′ will not be a reliable metric. In eq 7, the change in any two coupled states is expected to be equal and opposite. Consequently, and accounting for the underlying parabolic behavior of the ground state, we can rewrite the equation to depend on only one state,^68^
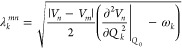
8The interstate coupling is then calculated by either eq 7 or eq 8 depending on how reliable the second derivatives are expected to be. Generally, we use eq 7 unless one of the states has no other couplings that could lead to a change in gradient. In addition, given that most states under consideration are degenerate, all the coupling terms are multiplied by a factor of 1/2, to avoid double counting.

### Quantum Dynamics

Quantum dynamics were performed using the Quantics 1.2^78^ implementation of the multiconfigurational time-dependent Hartree (MCTDH) method.^79,80^ The dynamics were performed using four model Hamiltonians. The first (model A) includes the two lowest singlet states and the eight lowest triplet states coupled with all terms described in this paper. Model B incorporates the same states, but removes the spin–orbit coupling gradients, i.e., the SOC is independent of *Q* and fixed to that at the FC geometry. Model C retains the SOC gradient, but removes the highest-lying triplet states (*T*_6_–*T*_8_). Finally, model D incorporates the same states as model A, but substitutes the state energies as calculated with EOM-CCSD.

Each normal mode in the model is described using 41 harmonic oscillator eigenfunction basis. To reduce the computational expense, mode pairs (ν_5_:ν_6_, ν_21_:ν_22_, ν_23_:ν_24_, ν_72_:ν_73_, and ν_19_:ν_50_) are contracted into a single degree of freedom. Simulations are run within the multistate formalism, and 12 single-particle functions are used for each singlet and triplet state. These ensure convergence during the simulations presented herein. The initial wave function is built using one-dimensional harmonic oscillator functions with zero initial momentum and is vertically excited into the *S*_1_ state at the FC geometry.

## Results

### Excited State Properties and Spin–Orbit Coupling of HTANGO

Table 1 shows the TD-DFT(PBE0) and EOM-CCSD computed excited state energies of HTANGO at critical geometries on the ground and excited state potential energy surfaces. At the ground state (*S*_0_) optimized geometry the lowest lying singlet and triplet states in TANGO are degenerate pairs of *E*′ symmetry, with the density difference associated with the *S*_1_ state shown in Figure 1b.

**Table 1:** Excited State Energies at the Ground State and Lowest Singlet and Triplet Excited States Optimized Geometries of HTANGO

	*S*_0_				*S*_1_		*T*_1_	
	TD-DFT		EOM-CCSD		TD-DFT		TD-DFT	
	*E*_*i*_ [eV]	*f*_*osc*_	*E*_*i*_ [eV]	*f*_*osc*_	*E*_*i*_ [eV]	*f*_*osc*_	*E*_*i*_ [eV]	*f*_*osc*_
*S*_1_(E′)	3.41	0.20	3.31	0.20	3.27	0.19	3.24	0.21
*S*_2_(E′)	3.41	0.20	3.30	0.20	3.38	0.21	3.33	0.19
*T*_1_(E′)	2.79	-	2.89	-	2.66	-	2.56	-
*T*_2_(E′)	2.79	-	2.89	-	2.73	-	2.71	-
*T*_3_(E″)	3.15	-	3.42	-	3.01	-	2.90	-
*T*_4_(E″)	3.15	-	3.41	-	3.10	-	3.06	-
*T*_5_(A_1_″)	3.30	-	3.52	-	3.21	-	3.16	-
*T*_6_(A_1_″)	3.32	-	3.14	-	3.19	-	3.14	-
*T*_7_(E″)	3.43	-	3.32	-	3.31	-	3.25	-
*T*_8_(E″)	3.43	-	3.30	-	3.39	-	3.34	-

At the ground state geometry, the lowest singlet states of HTANGO, computed using TD-DFT, are at 3.41 eV, which is in good agreement with EOM-CCSD but higher than the 3.10 eV (400 nm) observed in the experimental absorption spectrum in ref (41). Both the TD-DFT and EOM-CCSD calculations exhibit 8 triplet states below or within close proximity to the degenerate *S*_1_/*S*_2_ pair, which would be favorable for ISC. At the lowest singlet excited state optimized geometry, related to the fluorescence properties, the *S*_1_ state is at 3.27 eV, indicating a Stokes shift of 0.14 eV in good agreement with experiment. Despite the structural rearrangement, which lifts the degeneracy of the *S*_1_/*S*_2_ states, there remains a high density of triplet states lower than the *S*_1_ state. The lowest triplet state at the triplet geometry, corresponding to the phosphorescence properties, exhibits an energy of 2.56 eV, in excellent agreement with the experimentally recorded phosphorescence 475 nm (2.61 eV).

Table 2 shows the spin–orbit coupling matrix elements (SOCME) calculated between the low-lying excited states. As expected for a purely organic molecule, the coupling is generally weak with all couplings between singlet and triplet states ≤10 cm^–1^. While larger SOCME exist between the triplet states, these are all small when compared to the calculated diabatic couplings (λ, see Table S3) and will therefore not play a significant role during the dynamics. Table 3 compares the nonzero SOCME between the singlet and triplet states for HTANGO to both benzophenone and fluorenone. The SOCME of HTANGO are substantially smaller than those of benzophenone, due to the constrained planar structure,^49^ however, owing to the degeneracy of the *S*_1_ and *S*_2_ and the *T*_3_ and *T*_4_ states, HTANGO has six coupling pathways, whereas, for benzophenone, there are two. Consequently, while the SOCME are smaller in HTANGO than in benzophenone, the existence of three times as many pathways could be expected to somewhat compensate for the smaller SOCME.^36^ The SOCME in HTANGO is significantly higher than that of fluorenone. Consequently, while the rigid planarity of HTANGO clearly reduces the SOCME, compared to benzophenone, its high symmetry facilitates the multiple pathways which are likely to encourage ISC.

**Table 2:** All Non-zero Spin–Orbit Couplings in cm^–1^ at the Ground State Optimized Geometry of HTANGO

	*T*_2_	*T*_3_	*T*_4_	*T*_5_	*T*_6_	*T*_7_	*T*_8_
*S*_1_	-	6.4	6.2	10.3	-	-	-
*S*_2_	-	6.0	6.2	10.5	-	-	-
*T*_1_	0.1	15.8	15.4	22.5	-	0.1	-
*T*_2_	-	15.1	15.3	22.9	-	-	0.1
*T*_3_	-	-	2.8	-	29.4	9.2	9.2
*T*_4_	-	-	-	-	29.3	9.3	9.5
*T*_5_	-	-	-	-	0.3	8.2	7.9

**Table 3:** Summary of the SOC Pathways in the HTANGO Molecule Compared to Similar Molecules Studied by Karak *et al*.^49^

Molecule	Pathway	SOCME (cm^–1^)	Δ*E* (eV)
Benzophenone	*S*_1_ → *T*_2_	52.6^49^	0.14^49^
	*S*_1_ → *T*_1_	23.3^49^	0.62^49^
HTANGO	*S*_1_ → *T*_5_	10.3	0.11
	*S*_2_ → *T*_5_	10.5	0.11
	*S*_1_ → *T*_4_	6.2	0.26
	*S*_1_ → *T*_4_	6.2	0.26
	*S*_1_ → *T*_3_	6.4	0.26
	*S*_2_ → *T*_3_	6.0	0.26
Fluorenone	*S*_1_ → *T*_2_	0.56^49^	0.50^49^
	*S*_1_ → *T*_1_	2.10^49^	0.60^49^

### Developing the Linear Vibronic Coupling Hamiltonian

Having established the excited state properties, we now seek a more detailed insight into the excited state processes using quantum dynamics, which requires a Hamiltonian. HTANGO has 34 atoms and therefore 96 normal modes. Consequently, calculating the full PES is unrealistic. To reduce the computational effort we use a reduced space Hamiltonian, based upon the linear vibronic coupling model. The validity of the linear vibronic coupling Hamiltonian in this case is based upon the assumption that the rigidity of HTANGO means that anharmonicity of the potential energy surfaces does not play a significant role in the dynamics. To assess the accuracy, we use the recently introduced Global Anharmonicity Parameter^77^ (GAP), which in the *S*_1_ state is 5% and in the *T*_1_ state is 13%. A GAP of around 10% suggests that anharmonicity effects are small and the LVC potential can be considered a reliable approximation. While the *T*_1_ state slightly exceeds this value, we note that the majority of this is due to the effect of the JT coupling, which gives rise to a gradient at the FC geometry in the state energies and inflates the GAP.^77^ In addition, the geometries predicted by the LVC model are very similar to the fully optimized structures, with the RMSD of the *T*_1_ state being 0.007 Å.

**Figure 3:**
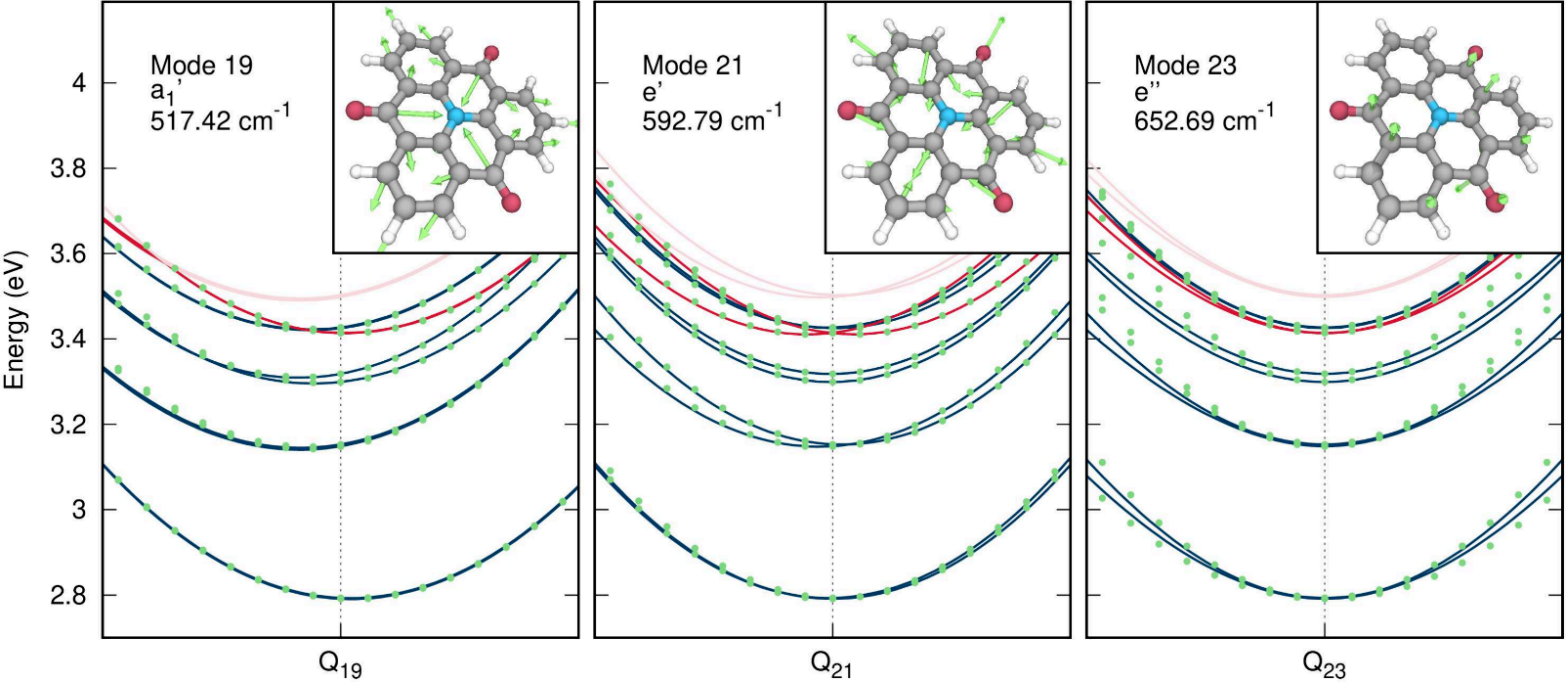
Energy of the singlet (red) and triplet (blue) states as the molecule is projected along representative modes considered in the dynamics in dimensionless normal coordinates (similar plots for the other modes used in the dynamics are shown in the SI). The green points represent the energy levels given by the LVC Hamiltonian along the normal modes. The inset image shows the mode on the molecule

Figure 3 shows cuts of the potential energy curves along selected modes, with the remaining modes used in the Hamiltonian included in Figure S1. The green points represent the potentials reproduced using the LVC parameters, while the blue and red lines represent the potentials calculated using TD-DFT(PBE0). Overall, these plots reveal good agreement between the LVC model and the TD-DFT calculations, indicating that the LVC represents a good approximation for the potential energy surface.

In total ten normal modes were considered important for the LVC Hamiltonian, an assessment which was based upon the magnitude of the first-order coupling terms (see Tables S4 and S5 in the Supporting Information) and these include three degenerate pairs and two fully symmetric modes. The two *a*_1_′ modes chosen are ν_19_ and ν_50_. ν_19_ has the largest intrastate couplings of any low-lying mode and the effect of this can be observed in Figure 3, which shows the excited state potentials shifted with respect to the ground state potential, illustrated with the dashed line. This shift is responsible for driving excited state dynamics as well as causing broadening, through vibronic structure, to the emission spectrum.^81^ In contrast, ν_50_ is chosen as it has the largest difference in intrastate couplings between the singlet pair and the energetically nearby triplet states, effectively lowering the energy gap upon distortion along this mode.

Two pairs of *e*′ modes are selected to allow for coupling within degenerate pairs and between the upper triplet states (see Figure 2). Modes ν_21_:ν_22_ are selected as they are the lowest lying *e*′ modes with a significant JT coupling. This can be observed in Figure 3 as the splitting of the degenerate states as you move away from the Franck–Condon geometry. ν_72_:ν_73_ were selected because, of all the *e*′ pairs, they induce the largest JT coupling (see SI). These modes also contribute a significant coupling between the high-lying triplet states as shown in Table 5 in the Supporting Information. Finally, to couple the high-lying triplet cluster to the lowest degenerate triplet pair, we use a pair of modes of *e*″ symmetry. Here, modes ν_5_:ν_6_ and ν_23_:ν_24_ were chosen because they have the largest average coupling of any low-lying mode.

### Quantum Dynamics

**Figure 4:**
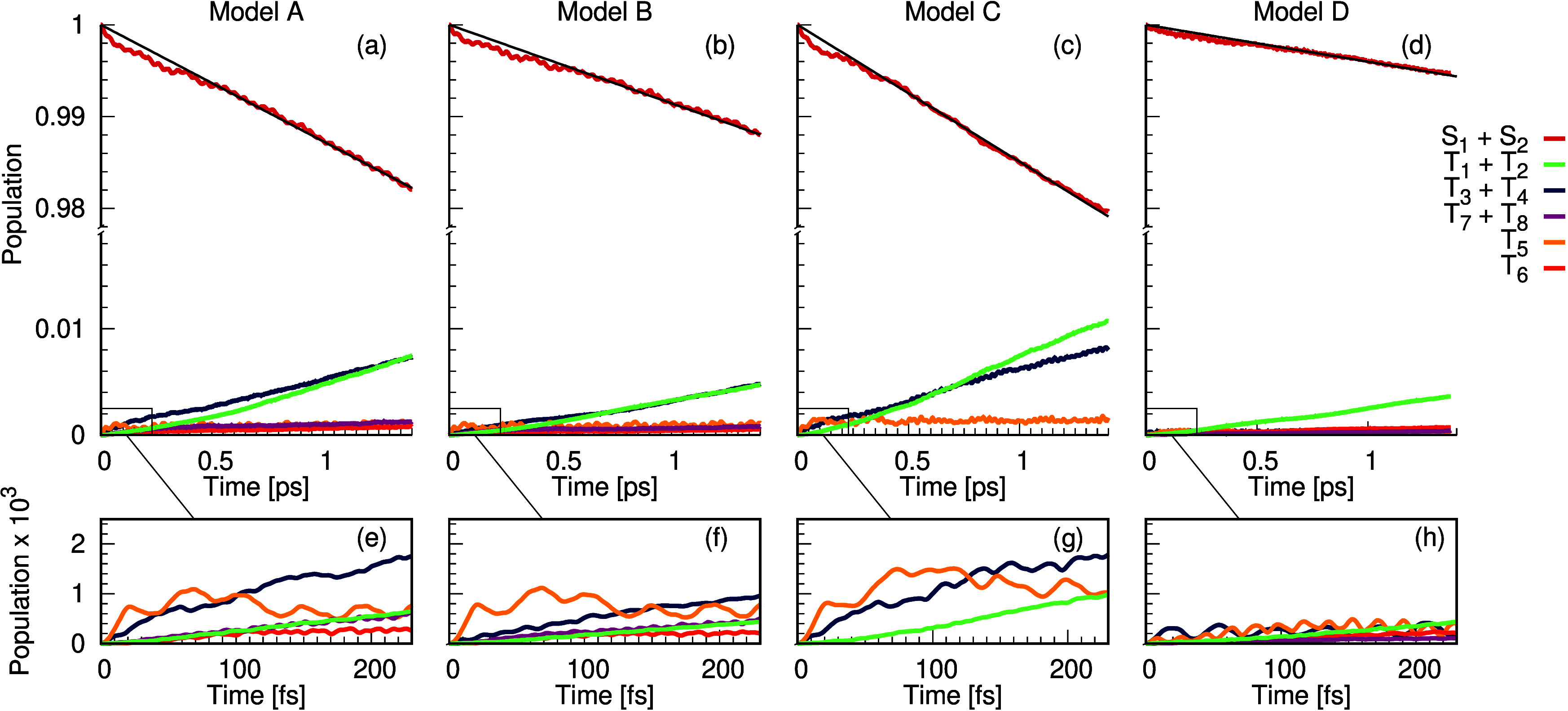
Population kinetics of the singlet and triplet states for the first 1.5 ps after excitation into *S*_1_ for the full model Hamiltonian (a), the case where SOC gradients have been removed (b), the case where the three highest lying triplet states have been removed (c), and the case where the EOM-CCSD state energies have been used in place of the TDDFT energies (d). The initial dynamics for the first 200 fs are shown in (e)–(h) for the same models, respectively.

Having developed the excited state potential, using the LVC Hamiltonian, we now use this to study the ISC dynamics of HTANGO. Figure 4a shows the population kinetics of the singlet and triplet states for the first 1.5 ps after excitation into the lowest singlet excited state (*S*_1_). This dynamics (Model A) includes all the couplings described in the previous sections. By fitting the decay of the population kinetic of the singlet states, we observe a *k*_*ISC*_^*A*^ = 1.3 × 10^10^ s^–1^, which is comparable to the *k*_*ISC*_ reported for benzophenone of ∼10^11^ s^–1^ and therefore very fast for an organic molecule. To elucidate the mechanism of the ISC, we modify this model to identify the most important pathways.

Figure 4b shows the same dynamics, but with the SOC gradients removed. This shows a reduction in the ISC rate, *k*_*ISC*_^*B*^ = 0.85 × 10^10^ s^–1^, highlighting that Herzbeg–Teller interactions play an important role. However, as the rate of ISC remains high, the primary mechanism for this intersystem crossing is the direct spin-allowed transitions. Figure 4c shows the dynamics for the model in which the three highest triplet states (*T*_6_, *T*_7_, and *T*_8_) have been removed. Here, the fitted intersystem crossing rate, *k*_*ISC*_^*C*^ = 1.5 × 10^10^ s^–1^, is similar to the model with all states included, suggesting that the higher lying states do not play a significant role in the dynamics. For all models, the population kinetics of the triplet states is shown for the first 200 fs in Figures 4e–h. The populations of *T*_3_, *T*_4_, and *T*_5_ rise the fastest in all cases as these are directly coupled and degenerate with the *S*_1_ and *S*_2_ states. The population then rises in the remaining triplet states as it transfers from the *T*_3_, *T*_4_, and *T*_5_ states via vibronic coupling.

**Figure 5:**
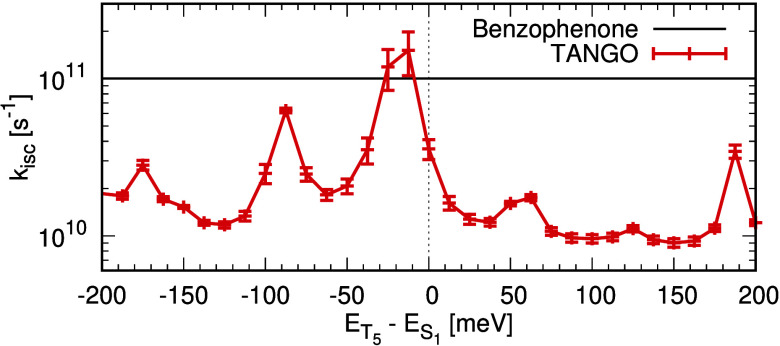
Intersystem-crossing rates calculated for the Model A Hamiltonian as a function of the energy difference between *T*_5_ and *S*_1_. For context, the *k*_ISC_ for benzophenone is shown as reported by Karak et al.^49^

The above dynamics reveal the importance of coupling to the *T*_5_ state. A key driving force for this is the close nature of the *S*_1_/*S*_2_ state with the *T*_5_ state, with the *T*_5_ appearing 0.11 eV below the singlet degenerate pair at the ground state geometry. However, as shown in Table 1, the ordering of the states changes in EOM-CCSD and the singlet states are closer to the states with weak or zero coupling. The *T*_5_ state (calculated with TDDFT) with the larger SOC now occurs 0.21 eV higher in energy than the singlet states when calculated using EOM-CCSD. As shown in Figure 4d (Model D) this reduces the population transfer and therefore the ISC rate to *k*_*ISC*_^*A*^ = 0.4 × 10^10^ s^–1^. To further study this effect, we calculated the population dynamics for a range of Hamiltonians where all the parameters are taken from the model A Hamiltonian with the exception of the energy of the *T*_5_ state, which is scanned from 0.2 eV below the energy of the lowest singlet pair to 0.2 eV above. Figure 5 shows the fitted ISC rates for the range of Hamiltonians. Here, we see that the ISC depends strongly on the relative energy of the *S*_1_ and *T*_5_ states and indeed, if the states are degenerate, the ISC is nearly an order of magnitude higher than that predicted by the TD-DFT and EOM-CCSD state energies. This demonstrates that relatively small changes in structure, which bring the energy of the *S*_1_ and *T*_5_ states closer together, can have a dramatic effect on the dynamics, and this was used as a design principle to enhance the ISC rate in these molecules. To illustrate the importance of subtle structural changes, *ab initio* molecular dynamics calculations performed in the electronic ground state (see Figure S8) show that even at 77 K, the difference in energy between the *T*_5_ and *S*_1_ can fluctuate significantly, with a standard deviation of 31 meV over a 1 ps range, meaning using thermal energy alone, the system has sufficient energy to traverse the potential reaching regions where the *T*_5_ and *S*_1_ states are close in energy.

### Electronic Structure of Dimer

The previous section has used quantum dynamics to investigate the ISC dynamics of the isolated monomer, highlighting an ISC rate comparable to benzophenone despite the rigidity of the structure. In ref (41), the authors demonstrated a significant difference between solution and crystal emission properties. Indeed, with low concentration in chloroform solution, the emission maximum is at ∼425 nm (2.92 eV) slightly lower than the values calculated above in Table 1 (3.27 eV). However, in the solid state the prompt and delayed emission maxima redshift to 550 nm (2.25 eV). This was proposed to be the formation of π-stack dimers which can be hindered using bulky substituents.

**Figure 6:**
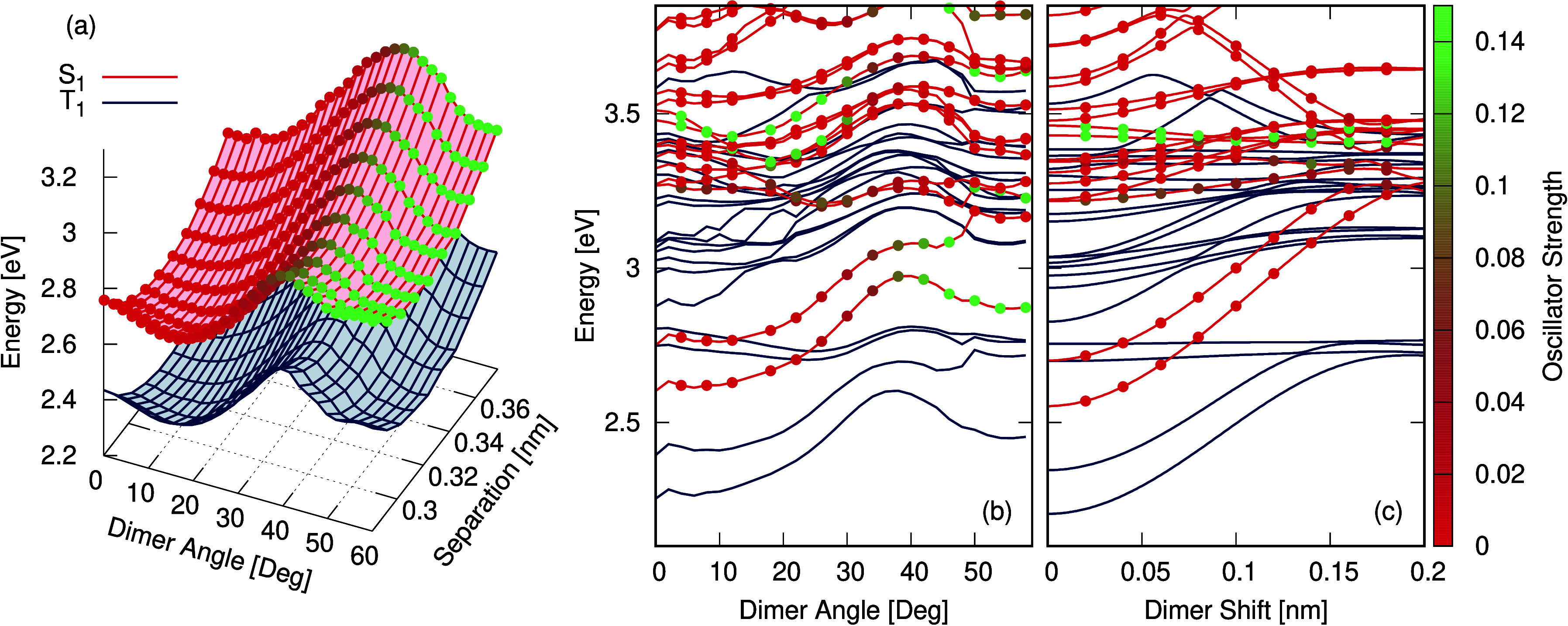
(a) Energy of the *S*_1_ (red) and *T*_1_ (blue) states for a range of dimer systems of varying angles and separation. In each system, the dimer angle and separation is constrained and the systems are otherwise optimized into the *S*_1_ geometry. (b) Energy of the low-lying singlet (red) and triplet (blue) states for a range of dimer systems with varying angles. The dimer angle is constrained and the systems are otherwise optimized into the *S*_1_ geometry. (c) Energy of the low-lying singlet (red) and triplet (blue) states for a range of dimer systems horizontally displaced from the low-angle minimum position. In all cases, the oscillator strength of the singlet states is shown as colored points on the surface, where red (green) points correspond to low (high) strengths.

To shed further light on the role of crystal packing on the excited state properties, Figure 6 shows the potential energy surfaces of the dimer around the excited *S*_1_ geometry as a function of the angle, distance, and horizontal displacement between each monomer unit with respect to the other, consequently addressing the energetic landscape of potential J-, H-, and X-aggregates in the present system.^82^ A schematic of the dimer structure used is shown in Figure S9. Figure 6a shows only the *S*_1_ and *T*_1_ states as a function of the angle and distance between the monomer units. These potentials exhibit two distinct minima in each state along dimer angles of 10° and 60°. The energy of the *T*_1_ state at 10° is 2.24 eV, in excellent agreement with the emission spectrum of 2.25 eV (550 nm) reported in ref (41). In addition, the spectra in ref (41) show a small shoulder at around 470 nm (2.63 eV), close to the 2.58 eV of the singlet state at 10°. While the former has a very small oscillator strength (indicated by colored dots) compared to the singlet state minimum at 60° it is the lowest energy state, and therefore after vibrational cooling, the molecule can reside in this minima from where it can either emit or undergo ISC. Indeed, assuming a thermal equilibrium between two *S*_1_ minima, the relative intensity of each band, at room temperature, would be
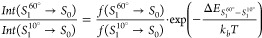
9Given the energy gap between the two minima of 0.26 eV, *f*(*S*_1_^10°^) = 0.00002 and *f*(*S*_1_^60°^) = 0.14, the ratio, , is 0.3 at 300 K, meaning emission from *S*_1_^10°^ will be about 3 times larger than *S*_1_^60°^. Importantly, the steady state emission spectra reported in ref (41) show two weak shoulders at 450 and 470 nm, corresponding to 2.76 and 2.63 eV. These exhibit an ∼1:3 ratio, consistent with the above analysis, and we therefore assign them to the *S*_1_^60°^ and *S*_1_^10°^, respectively.

The shape of the dimer potential energy surface and the oscillator strength increasing with the angle between the monomer units can be further understood in Figure 6b, which shows all of the low-lying excited states in a one-dimensional cut. This shows that along the dimer angle, the low-lying singlet states intersect the higher-lying triplet states, which would be expected to enhance ISC.^83^ This combined with the rigidity of the structure leading to a low nonradiative rate will be a strong contributing factor to the high phosphorescence yield, supporting the observations made by Hamzehpoor *et al*.^41^ In addition, this clearly shows that although the rotated dimers (X-aggregate) have the largest oscillator strength, the lower energy structure is the structure for the 10°, *pseudo* H-aggregate, which is at 2.58 eV while the higher energy minimum at 60° is at 2.80 eV (443 nm). A similar thing is observed for the lowest triplet states which are at 2.3 eV (539 nm) at 10° and 2.48 eV (517 nm) for 60°. Finally, Figure 6c shows the potentials along the horizontal displacement between the two dimer units, i.e., differentiating between J- and H-aggregation. This clearly shows for the *S*_1_ and *T*_1_ states that a single minimum for the 0 dimer shift is observed, i.e., the excited state favors H-aggregation. Interestingly, the *T*_3_ and *T*_4_ excited states exhibit very little dependence on this shift leading to another crossing between these states and the lowest two singlet states.

In the solid state, the presence of monomeric states as well as dimeric (and potentially high-order) states competing with the multitude of close lying singlet and triplet states which both have multiple emissive configurations (*i.e., rotation of dimer angles*) means the solid state excited state dynamics of this system is likely to be complicated with many competing pathways. However, overall, the present simulations highlight the importance of intramolecular interactions for describing the phosphorescence properties, consistent with ref (24), which illustrated the special role of molecular dimers in persistent RTP emission.

## Discussion and Conclusions

In this work we have performed a detailed computational investigation into the excited state properties of the planar *D*_3*h*_ symmetric azatriangulenetrione, HTANGO, with the focus of understanding the ISC and effect of intramolecular interactions which influences its performance as an organic room temperature phosphorescent (ORTP) dye.

Our quantum dynamics have demonstrated that HTANGO exhibits fast intersystem crossing (ISC) with a rate comparable to benzophenone. While the planar rigid structure of HTANGO appears to reduce the SOCME, compared to benzophenone, the high symmetry gives rise to a high density of triplet excited states in close proximity to the lowest singlet states, facilitating rapid population transfer. Among all of these states, the proximity of the *T*_5_ state to *S*_1_ in particular is identified as most significant to the rapid ISC.

Using quantum chemistry simulations of a model dimer system, our study has shed light on the solid-state emission properties of HTANGO. We have found that the emission maximum in the solid state is red-shifted compared to the solution phase, indicating the formation of π-stack dimers. The potential energy surfaces of these dimers reveal two distinct minima in the excited singlet (*S*_1_) and triplet (*T*_1_) states at angles of 10° and 60°. The energy of the *T*_1_ state at 10° agrees well with the reported emission spectrum, highlighting the influence of crystal packing on the excited state properties.

Overall, our computational investigation provides valuable insights into the excited state properties of HTANGO, offering a deeper understanding of its potential as an ORTP dye and the influence of crystal packing on its emission characteristics.

## Supporting Information Available

Computational details, Hamiltonian parameters, plots of the singlet and triplet potential energy surfaces, and molecular dynamics (PDF)
