# Supplementary material for: Spin-Vibronic Intersystem Crossing and Molecular Packing Effects in Heavy Atom Free Organic Phosphor
Source: J Chem Theory Comput. 2024 Jan 25;20(3):1337–46. doi: 10.1021/acs.jctc.3c01220 (PMC10867843; doi:10.1021/acs.jctc.3c01220)
Supplement: Supplementary file 1 — ct3c01220_si_001.pdf [file ct3c01220_si_001.pdf]

# Supporting Information: Spin-Vibronic Intersystem Crossing and Molecular Packing Effects in Heavy Atom Free Organic Phosphor

Thomas Pope,† Julien Eng,† Andrew Monkman,‡ and Thomas J Penfold*,†

†*Chemistry, School of Natural and Environmental Sciences, Newcastle University, Newcastle upon Tyne, NE1 7RU, UK*

‡*Department of Physics, Durham University, South Road, Durham, DH1 3LE, UK*

E-mail: tom.penfold@newcastle.ac.uk

## List of Tables

S1 Computational details for the MCTDH simulations of the LVC model presented in this work. $N_i$,$N_j$ are the number of primitive harmonic oscillator discrete variable representation (DVR) basis functions used to describe each mode. $n_{\text{SPF}}$ is the number of single-particle functions used to describe the wavepacket on each state. The CPU time is reported for single core calculations on an AMD Ryzen threadripper 3970x processor for the first 1.5ps of the dynamics of the given model Hamiltonian. . . . . . . . . . . . . . . . . . . 4

S2 SOCME parameters for the Full Hamiltonian in $\text{cm}^{-1}$, where $\dot{\eta}_k^{mn}$ is the gradient of the SOCME along mode $k$. . . . . . . . . . . . . . . . . . . . . . . 5

S3 LVC parameters along all the modes included in the Full Model Hamiltonian in meV . . . . . . . . . . . . . . . . . . . . . . . . . . . . . . . . . . 6

S4 Full list of the normal modes of HTANGO with symmetry and energy . . . . 7
S5 Intrastate Coupling Parameters along all fully symmetric modes in meV. . . 7
S6 Coupling parameters along all $e'$ mode pairs from $\nu_8$ to $\nu_{63}$ in meV. . . . . . 8
S7 Coupling parameters along all $e'$ mode pairs from $\nu_{64}$ to $\nu_{77}$ in meV. . . . . . 9
S8 Coupling parameters along all $e''$ mode pairs in meV. . . . . . . . . . . . . . 10
S9 All non-zero spin-orbit couplings in $cm^{-1}$ at the ground state geometry . . . 11

## List of Figures

S1 Energy of the Singlet (Red) and Triplet (Blue) states as the molecule is projected along all modes considered in the Dynamics. The green points represent the energy levels given by the LVC Hamiltonian along the normal modes. The inset image shows the mode on the molecule . . . . . . . . . . . . . . . . . . . 12
S2 Energy of the Singlet (Red) and Triplet (Blue) states as the molecule is projected along modes 1 to 15. The inset image shows the mode on the molecule 13
S3 Energy of the Singlet (Red) and Triplet (Blue) states as the molecule is projected along modes 16 to 30. The inset image shows the mode on the molecule 14
S4 Energy of the Singlet (Red) and Triplet (Blue) states as the molecule is projected along modes 31 to 45. The inset image shows the mode on the molecule 15
S5 Energy of the Singlet (Red) and Triplet (Blue) states as the molecule is projected along modes 46 to 60. The inset image shows the mode on the molecule 16
S6 Energy of the Singlet (Red) and Triplet (Blue) states as the molecule is projected along modes 61 to 75. The inset image shows the mode on the molecule 17
S7 Energy of the Singlet (Red) and Triplet (Blue) states as the molecule is projected along modes 76 to 90. The inset image shows the mode on the molecule 18

S8 Ab-Initio Molecular Dynamics on a TANGO monomer with a time-step of 0.5fs. The temperature is maintained at 77K via a Berendsen thermostat with a time constant of 10fs. Throughout the simulation, the temperature of the system has a standard deviation of 7K arount 77K. (a) Singlet (red) and Triplet (blue) state energies as a function of time. Highlighted are the $S_1$ and $T_5$ energies. (b) Histogram of the $S_1$ (red) and $T_5$ (blue) energies throughout the MD run. (c) Difference between the $T_5$ and $S_1$ energies as a function of time. (d) Histogram of the difference between the $S_1$ and $T_5$ energies throughout the MD run. Here, the standard deviation is calculated to be 31 meV. . . . . . . . . . . . . . . . . . . . . . . . . . . . . . . . . . . . 19

S9 Schematic of the TANGO Dimer system, showing the dimer spacing (red) and dimer angle (green) parameters. . . . . . . . . . . . . . . . . . . . . . . . . . . . 20

Table S1: Computational details for the MCTDH simulations of the LVC model presented in this work. $N_i$,$N_j$ are the number of primitive harmonic oscillator discrete variable representation (DVR) basis functions used to describe each mode. $n_{\mathrm{SPF}}$ is the number of single-particle functions used to describe the wavepacket on each state. The CPU time is reported for single core calculations on an AMD Ryzen threadripper 3970x processor for the first 1.5ps of the dynamics of the given model Hamiltonian.

| Model | Modes | | $N_i$ | $N_j$ | $n_{\mathrm{SPF}}$ | | | | | | | | | | CPU time (d:h:m:s) |
|---|---|---|---|---|---|---|---|---|---|---|---|---|---|---|---|
| | | | | | $S_1$ | $S_2$ | $T_1$ | $T_2$ | $T_3$ | $T_4$ | $T_5$ | $T_6$ | $T_7$ | $T_8$ | |
| A | $\nu_5$ | $\nu_6$ | 41 | 41 | 12 | 12 | 12 | 12 | 12 | 12 | 12 | 12 | 12 | 12 | 4 : 13 : 13 : 33 |
| | $\nu_{21}$ | $\nu_{22}$ | 41 | 41 | 12 | 12 | 12 | 12 | 12 | 12 | 12 | 12 | 12 | 12 | |
| | $\nu_{23}$ | $\nu_{24}$ | 41 | 41 | 12 | 12 | 12 | 12 | 12 | 12 | 12 | 12 | 12 | 12 | |
| | $\nu_{72}$ | $\nu_{73}$ | 41 | 41 | 12 | 12 | 12 | 12 | 12 | 12 | 12 | 12 | 12 | 12 | |
| | $\nu_{19}$ | $\nu_{50}$ | 41 | 41 | 12 | 12 | 12 | 12 | 12 | 12 | 12 | 12 | 12 | 12 | |
| B | $\nu_5$ | $\nu_6$ | 41 | 41 | 12 | 12 | 12 | 12 | 12 | 12 | 12 | 12 | 12 | 12 | 1 : 06 : 15 : 33 |
| | $\nu_{21}$ | $\nu_{22}$ | 41 | 41 | 12 | 12 | 12 | 12 | 12 | 12 | 12 | 12 | 12 | 12 | |
| | $\nu_{23}$ | $\nu_{24}$ | 41 | 41 | 12 | 12 | 12 | 12 | 12 | 12 | 12 | 12 | 12 | 12 | |
| | $\nu_{72}$ | $\nu_{73}$ | 41 | 41 | 12 | 12 | 12 | 12 | 12 | 12 | 12 | 12 | 12 | 12 | |
| | $\nu_{19}$ | $\nu_{50}$ | 41 | 41 | 12 | 12 | 12 | 12 | 12 | 12 | 12 | 12 | 12 | 12 | |
| C | $\nu_5$ | $\nu_6$ | 41 | 41 | 12 | 12 | 12 | 12 | 12 | 12 | 12 | - | - | - | 2 : 14 : 35 : 13 |
| | $\nu_{21}$ | $\nu_{22}$ | 41 | 41 | 12 | 12 | 12 | 12 | 12 | 12 | 12 | - | - | - | |
| | $\nu_{23}$ | $\nu_{24}$ | 41 | 41 | 12 | 12 | 12 | 12 | 12 | 12 | 12 | - | - | - | |
| | $\nu_{72}$ | $\nu_{73}$ | 41 | 41 | 12 | 12 | 12 | 12 | 12 | 12 | 12 | - | - | - | |
| | $\nu_{19}$ | $\nu_{50}$ | 41 | 41 | 12 | 12 | 12 | 12 | 12 | 12 | 12 | - | - | - | |
| D | $\nu_5$ | $\nu_6$ | 41 | 41 | 12 | 12 | 12 | 12 | 12 | 12 | 12 | 12 | 12 | 12 | 4 : 09 : 16 : 58 |
| | $\nu_{21}$ | $\nu_{22}$ | 41 | 41 | 12 | 12 | 12 | 12 | 12 | 12 | 12 | 12 | 12 | 12 | |
| | $\nu_{23}$ | $\nu_{24}$ | 41 | 41 | 12 | 12 | 12 | 12 | 12 | 12 | 12 | 12 | 12 | 12 | |
| | $\nu_{72}$ | $\nu_{73}$ | 41 | 41 | 12 | 12 | 12 | 12 | 12 | 12 | 12 | 12 | 12 | 12 | |
| | $\nu_{19}$ | $\nu_{50}$ | 41 | 41 | 12 | 12 | 12 | 12 | 12 | 12 | 12 | 12 | 12 | 12 | |

Table S2: SOCME parameters for the Full Hamiltonian in cm$^{-1}$, where $\dot{\eta}_k^{mn}$ is the gradient of the SOCME along mode $k$.

| | $\dot{\eta}_k^{S_1\to T_3}$ | $\dot{\eta}_k^{S_1\to T_4}$ | $\dot{\eta}_k^{S_1\to T_5}$ | $\dot{\eta}_k^{S_2\to T_3}$ | $\dot{\eta}_k^{S_2\to T_4}$ | $\dot{\eta}_k^{S_2\to T_5}$ | $\dot{\eta}_k^{T_1\to T_3}$ |
|---|---|---|---|---|---|---|---|
| $\nu_{21}$ | -1.67 | - | 0.45 | 0.92 | 0.67 | -0.42 | 1.19 |
| $\nu_{22}$ | -1.16 | -0.44 | 0.44 | 0.43 | 1.17 | -0.44 | - |
| $\nu_{72}$ | -3.37 | -0.36 | 0.99 | 3.68 | 0.17 | -0.85 | -10.97 |
| $\nu_{73}$ | 1.45 | 2.22 | -0.93 | -1.78 | -1.93 | 0.91 | 4.57 |
| | $\dot{\eta}_k^{T_1\to T_4}$ | $\dot{\eta}_k^{T_1\to T_5}$ | $\dot{\eta}_k^{T_2\to T_3}$ | $\dot{\eta}_k^{T_2\to T_4}$ | $\dot{\eta}_k^{T_2\to T_5}$ | $\dot{\eta}_k^{T_3\to T_6}$ | $\dot{\eta}_k^{T_3\to T_7}$ |
| $\nu_{21}$ | - | 1.96 | 1.04 | -1.13 | - | 1.19 | - |
| $\nu_{22}$ | - | - | - | - | - | 1.11 | - |
| $\nu_{72}$ | -0.36 | -0.36 | 7.81 | - | -3.10 | -7.02 | 3.30 |
| $\nu_{73}$ | 2.91 | -3.39 | -1.39 | -6.23 | 3.31 | 6.39 | -8.64 |
| | $\dot{\eta}_k^{T_3\to T_8}$ | $\dot{\eta}_k^{T_3\to T_6}$ | $\dot{\eta}_k^{T_3\to T_7}$ | $\dot{\eta}_k^{T_3\to T_8}$ | $\dot{\eta}_k^{T_3\to T_6}$ | $\dot{\eta}_k^{T_3\to T_7}$ | $\dot{\eta}_k^{T_3\to T_8}$ |
| $\nu_{21}$ | -2.77 | -1.18 | - | 2.59 | - | -1.42 | 1.43 |
| $\nu_{22}$ | -1.05 | -1.11 | - | 1.05 | - | - | - |
| $\nu_{72}$ | 19.82 | 5.82 | - | -22.10 | -2.33 | -2.40 | 2.35 |
| $\nu_{73}$ | -11.28 | -6.83 | 9.19 | 11.26 | - | -2.50 | 2.55 |

Table S3: LVC parameters along all the modes included in the Full Model Hamiltonian in meV

| | $\lambda_k^{T_3\to T_5}$ | $\lambda_k^{T_4\to T_5}$ | $\lambda_k^{T_7\to T_5}$ | $\lambda_k^{T_8\to T_5}$ | $\lambda_k^{T_3\to T_6}$ | $\lambda_k^{T_4\to T_6}$ | $\lambda_k^{T_7\to T_6}$ | $\lambda_k^{T_8\to T_6}$ |
|---|---|---|---|---|---|---|---|---|
| $\nu_{21}$ | 6.59 | 5.24 | 3.88 | 3.91 | 5.39 | 3.44 | 3.48 | 3.51 |
| $\nu_{22}$ | 9.87 | 12.70 | 3.09 | 3.11 | 11.36 | 12.79 | 4.04 | 4.08 |
| $\nu_{72}$ | 48.99 | 38.90 | 18.80 | 18.95 | 28.53 | 59.52 | 45.57 | 46.00 |
| $\nu_{73}$ | 69.99 | 62.95 | 18.35 | 18.49 | 58.73 | 80.53 | 48.11 | 48.56 |

| | $\gamma_k^{S_1\to S_2}$ | $\gamma_k^{T_1\to T_2}$ | $\gamma_k^{T_3\to T_4}$ | $\gamma_k^{T_7\to T_8}$ |
|---|---|---|---|---|
| $\nu_{21}$, $\nu_{22}$ | 21.49 | 3.32 | 10.73 | 5.06 |
| $\nu_{72}$, $\nu_{73}$ | 61.77 | 64.40 | 46.55 | 100.68 |

| | $\lambda_k^{T_1\to T_3}$ | $\lambda_k^{T_1\to T_4}$ | $\lambda_k^{T_1\to T_5}$ | $\lambda_k^{T_1\to T_6}$ | $\lambda_k^{T_2\to T_3}$ | $\lambda_k^{T_2\to T_4}$ | $\lambda_k^{T_2\to T_5}$ | $\lambda_k^{T_2\to T_6}$ |
|---|---|---|---|---|---|---|---|---|
| $\nu_5$ | 9.19 | 9.25 | 10.96 | 11.16 | 17.72 | 17.84 | 21.15 | 21.55 |
| $\nu_6$ | 17.84 | 17.97 | 21.28 | 21.68 | 12.66 | 12.75 | 15.11 | 15.39 |
| $\nu_{23}$ | 14.05 | 14.15 | 16.76 | 17.07 | 19.93 | 20.07 | 23.80 | 24.24 |
| $\nu_{24}$ | 20.09 | 20.23 | 23.97 | 24.41 | 13.87 | 13.97 | 16.56 | 16.87 |

| | $\kappa_k^{S_1}$ | $\kappa_k^{S_2}$ | $\kappa_k^{T_1}$ | $\kappa_k^{T_2}$ | $\kappa_k^{T_3}$ | $\kappa_k^{T_4}$ | $\kappa_k^{T_5}$ | $\kappa_k^{T_6}$ |
|---|---|---|---|---|---|---|---|---|
| $\nu_{19}$ | -5.67 | -5.29 | -9.16 | -9.04 | 26.66 | 26.28 | 15.94 | 31.34 |
| $\nu_{50}$ | -0.75 | -1.26 | -8.13 | -9.07 | -47.24 | -48.41 | -40.33 | -63.08 |

| | $\kappa_k^{T_7}$ | $\kappa_k^{T_8}$ |
|---|---|---|
| $\nu_{19}$ | 22.46 | 22.29 |
| $\nu_{50}$ | -73.17 | -72.89 |

Table S4: Full list of the normal modes of HTANGO with symmetry and energy

| Mode | $\Gamma$ | $\omega[\mathrm{cm}^{-1}]$ | Mode | $\Gamma$ | $\omega[\mathrm{cm}^{-1}]$ | Mode | $\Gamma$ | $\omega[\mathrm{cm}^{-1}]$ | Mode | $\Gamma$ | $\omega[\mathrm{cm}^{-1}]$ |
|---|---|---|---|---|---|---|---|---|---|---|---|
| 1 | $e''$ | 80.7 | 25 | $e'$ | 692.4 | 49 | $a_2'$ | 1191.4 | 73 | $e'$ | 1665.8 |
| 2 | $e''$ | 84.1 | 26 | $e'$ | 693.2 | 50 | $a_1'$ | 1253.2 | 74 | $e'$ | 1675.7 |
| 3 | $a_2''$ | 134.0 | 27 | $a_2'$ | 716.5 | 51 | $e''$ | 1261.2 | 75 | $e'$ | 1678.6 |
| 4 | $a_2''$ | 169.5 | 28 | $a_1'$ | 801.2 | 52 | $e''$ | 1264.8 | 76 | $e'$ | 1719.0 |
| 5 | $e''$ | 268.0 | 29 | $a_2''$ | 807.7 | 53 | $e'$ | 1287.7 | 77 | $e'$ | 1720.3 |
| 6 | $e''$ | 270.6 | 30 | $e''$ | 817.9 | 54 | $e'$ | 1290.1 | 78 | $a_1'$ | 1742.7 |
| 7 | $a_1''$ | 273.1 | 31 | $e''$ | 818.6 | 55 | $a_1'$ | 1295.3 | 79 | $a_1''$ | 1852.6 |
| 8 | $e'$ | 312.1 | 32 | $e'$ | 845.3 | 56 | $e'$ | 1345.0 | 80 | $a_2''$ | 1865.2 |
| 9 | $e'$ | 313.3 | 33 | $e'$ | 845.7 | 57 | $e'$ | 1346.2 | 81 | $e''$ | 1888.3 |
| 10 | $e'$ | 351.7 | 34 | $a_2''$ | 923.2 | 58 | $a_2'$ | 1373.4 | 82 | $e''$ | 1890.5 |
| 11 | $e'$ | 351.7 | 35 | $a_1''$ | 956.6 | 59 | $a_2'$ | 1403.5 | 83 | $e''$ | 1934.9 |
| 12 | $a_2'$ | 362.7 | 36 | $e'$ | 974.4 | 60 | $e'$ | 1406.1 | 84 | $e''$ | 1936.1 |
| 13 | $e'$ | 371.2 | 37 | $e'$ | 974.4 | 61 | $e'$ | 1406.5 | 85 | $e''$ | 3096.8 |
| 14 | $e'$ | 371.7 | 38 | $e'$ | 1040.5 | 62 | $e'$ | 1422.5 | 86 | $e''$ | 3103.0 |
| 15 | $e''$ | 386.4 | 39 | $e'$ | 1040.5 | 63 | $e'$ | 1423.2 | 87 | $a_2''$ | 3177.4 |
| 16 | $e''$ | 388.8 | 40 | $a_2'$ | 1080.3 | 64 | $e'$ | 1491.1 | 88 | $e'$ | 3221.8 |
| 17 | $a_1'$ | 437.9 | 41 | $a_1'$ | 1129.0 | 65 | $e'$ | 1491.8 | 89 | $e'$ | 3222.8 |
| 18 | $a_2''$ | 481.4 | 42 | $e'$ | 1148.6 | 66 | $a_1'$ | 1518.2 | 90 | $e'$ | 3222.9 |
| 19 | $a_1'$ | 517.4 | 43 | $e'$ | 1149.4 | 67 | $a_2'$ | 1530.3 | 91 | $e'$ | 3231.0 |
| 20 | $a_2'$ | 522.0 | 44 | $a_2''$ | 1177.7 | 68 | $e'$ | 1538.6 | 92 | $e'$ | 3232.1 |
| 21 | $e'$ | 592.8 | 45 | $e''$ | 1179.2 | 69 | $e'$ | 1538.7 | 93 | $e'$ | 3232.2 |
| 22 | $e'$ | 593.9 | 46 | $e''$ | 1179.7 | 70 | $a_2'$ | 1636.7 | 94 | $e'$ | 3238.4 |
| 23 | $e''$ | 652.7 | 47 | $e'$ | 1189.1 | 71 | $a_1'$ | 1662.7 | 95 | $e'$ | 3239.2 |
| 24 | $e''$ | 652.7 | 48 | $e'$ | 1189.9 | 72 | $e'$ | 1665.5 | 96 | $e'$ | 3239.2 |

Table S5: Intrastate Coupling Parameters along all fully symmetric modes in meV.

| | $\kappa_k^{S_1}$ | $\kappa_k^{S_2}$ | $\kappa_k^{T_1}$ | $\kappa_k^{T_2}$ | $\kappa_k^{T_3}$ | $\kappa_k^{T_4}$ | $\kappa_k^{T_5}$ | $\kappa_k^{T_6}$ | $\kappa_k^{T_7}$ | $\kappa_k^{T_8}$ |
|---|---|---|---|---|---|---|---|---|---|---|
| $\nu_{17}$ | -9.61 | -9.54 | -6.28 | -6.23 | 10.27 | 10.07 | 9.23 | -5.30 | -12.92 | -12.85 |
| $\nu_{19}$ | -5.67 | -5.29 | -9.16 | -9.04 | 26.66 | 26.28 | 15.94 | 31.34 | 22.46 | 22.29 |
| $\nu_{28}$ | 45.82 | 45.76 | 49.33 | 49.24 | 31.82 | 31.51 | 35.45 | 14.34 | 3.64 | 4.07 |
| $\nu_{41}$ | 27.81 | 27.56 | 26.63 | 26.70 | 8.48 | 7.95 | 7.41 | 30.44 | 41.92 | 41.65 |
| $\nu_{50}$ | -0.75 | -1.26 | -8.13 | -9.07 | -47.24 | -48.41 | -40.33 | -63.08 | -73.17 | -72.89 |
| $\nu_{55}$ | -26.31 | -31.27 | -33.68 | -37.88 | -14.04 | -19.55 | -21.31 | -44.43 | -49.34 | -44.90 |
| $\nu_{66}$ | -15.10 | -16.02 | -2.01 | -2.97 | -8.12 | -8.71 | -15.60 | -26.50 | -17.79 | -18.92 |
| $\nu_{71}$ | -51.24 | -38.08 | -62.56 | -48.27 | -94.68 | -84.20 | -99.97 | -164.76 | -144.62 | -125.18 |

| | $\lambda_k^{T_3\to T_5}$ | $\lambda_k^{T_4\to T_5}$ | $\lambda_k^{T_7\to T_5}$ | $\lambda_k^{T_8\to T_5}$ | $\lambda_k^{T_3\to T_6}$ | $\lambda_k^{T_4\to T_6}$ | $\lambda_k^{T_7\to T_6}$ | $\lambda_k^{T_8\to T_6}$ | $\gamma_k^{S_1\to S_2}$ | $\gamma_k^{T_1\to T_2}$ | $\gamma_k^{T_3\to T_4}$ | $\gamma_k^{T_7\to T_8}$ |
|---|---|---|---|---|---|---|---|---|---|---|---|---|
| $\nu_8$ | 4.75 | 2.70 | 4.04 | 4.08 | 3.11 | 6.51 | 7.62 | 7.69 | 1.49 | 1.42 | 7.89 | 13.26 |
| $\nu_9$ | 9.84 | 10.50 | 4.41 | 4.45 | 11.98 | 9.55 | 7.75 | 7.82 | | | | |
| $\nu_{10}$ | 7.11 | 5.92 | 3.93 | 3.96 | 6.11 | 7.66 | 6.15 | 6.21 | 5.94 | 4.08 | 6.29 | 4.54 |
| $\nu_{11}$ | 6.55 | 7.43 | 3.43 | 3.46 | 8.25 | 6.58 | 5.90 | 5.95 | | | | |
| $\nu_{13}$ | 9.01 | 8.71 | 7.92 | 7.98 | 13.99 | 3.94 | 13.59 | 13.71 | 6.83 | 5.72 | 7.56 | 13.35 |
| $\nu_{14}$ | 8.32 | 8.04 | 7.83 | 7.89 | 6.02 | 13.56 | 13.99 | 14.12 | | | | |
| $\nu_{21}$ | 6.59 | 5.24 | 3.88 | 3.91 | 5.39 | 3.44 | 3.48 | 3.51 | 21.49 | 3.32 | 10.73 | 5.06 |
| $\nu_{22}$ | 9.87 | 12.70 | 3.09 | 3.11 | 11.36 | 12.79 | 4.04 | 4.08 | | | | |
| $\nu_{25}$ | 0.88 | 2.56 | 4.20 | 4.23 | 4.12 | 3.16 | 6.12 | 6.18 | 13.86 | 10.26 | 3.80 | 19.43 |
| $\nu_{26}$ | 5.60 | 4.81 | 4.39 | 4.42 | 3.89 | 6.76 | 6.46 | 6.52 | | | | |
| $\nu_{32}$ | 3.86 | 2.18 | 7.90 | 7.97 | 11.61 | 11.90 | 15.61 | 15.76 | 5.74 | 3.22 | 1.63 | 39.41 |
| $\nu_{33}$ | 5.39 | 2.66 | 7.80 | 7.86 | 11.37 | 12.85 | 15.99 | 16.13 | | | | |
| $\nu_{36}$ | 24.22 | 19.50 | 9.20 | 9.28 | 23.77 | 22.85 | 6.92 | 6.98 | 16.43 | 17.08 | 17.61 | 7.39 |
| $\nu_{37}$ | 33.65 | 30.08 | 9.34 | 9.42 | 34.31 | 33.43 | 6.99 | 7.06 | | | | |
| $\nu_{38}$ | 33.30 | 30.98 | 3.10 | 3.13 | 23.93 | 41.73 | 29.04 | 29.31 | 14.45 | 13.50 | 19.91 | 30.41 |
| $\nu_{39}$ | 6.58 | 7.99 | 2.05 | 2.07 | 24.62 | 23.74 | 28.65 | 28.92 | | | | |
| $\nu_{42}$ | 5.49 | 9.04 | 7.64 | 7.70 | 9.46 | 6.19 | 4.52 | 4.57 | 1.92 | 1.16 | 11.15 | 8.91 |
| $\nu_{43}$ | 20.64 | 18.91 | 7.38 | 7.44 | 20.54 | 21.44 | 5.10 | 5.15 | | | | |
| $\nu_{47}$ | 11.41 | 10.23 | 4.82 | 4.86 | 5.17 | 15.32 | 11.46 | 11.57 | 9.04 | 4.26 | 4.59 | 8.17 |
| $\nu_{48}$ | 7.15 | 5.59 | 4.17 | 4.20 | 7.69 | 12.18 | 11.49 | 11.60 | | | | |
| $\nu_{53}$ | 10.03 | 8.08 | 9.34 | 9.42 | 4.02 | 4.54 | 6.94 | 7.01 | 20.00 | 19.59 | 19.58 | 20.99 |
| $\nu_{54}$ | 29.69 | 26.08 | 9.09 | 9.16 | 29.84 | 29.45 | 7.62 | 7.69 | | | | |
| $\nu_{56}$ | 11.87 | 11.12 | 5.02 | 5.06 | 21.56 | 27.30 | 28.40 | 28.66 | 9.55 | 35.46 | 10.23 | 32.53 |
| $\nu_{57}$ | 20.88 | 20.18 | 4.95 | 4.99 | 12.45 | 32.97 | 28.88 | 29.14 | | | | |
| $\nu_{60}$ | 3.64 | 9.96 | 8.32 | 8.39 | 6.24 | 12.82 | 11.25 | 11.35 | 16.45 | 12.36 | 3.20 | 2.54 |
| $\nu_{61}$ | 10.48 | 4.00 | 8.50 | 8.57 | 12.78 | 4.52 | 10.55 | 10.65 | | | | |
| $\nu_{62}$ | 16.65 | 15.64 | 9.20 | 9.27 | 16.84 | 17.44 | 10.38 | 10.48 | 5.26 | 8.90 | 12.55 | 12.01 |
| $\nu_{63}$ | 22.18 | 20.95 | 9.14 | 9.21 | 22.83 | 22.97 | 10.59 | 10.69 | | | | |

Table S6: Coupling parameters along all $e'$ mode pairs from $\nu_8$ to $\nu_{63}$ in meV.

| | $\lambda_k^{T_3\to T_5}$ | $\lambda_k^{T_4\to T_5}$ | $\lambda_k^{T_7\to T_5}$ | $\lambda_k^{T_8\to T_5}$ | $\lambda_k^{T_3\to T_6}$ | $\lambda_k^{T_4\to T_6}$ | $\lambda_k^{T_7\to T_6}$ | $\lambda_k^{T_8\to T_6}$ | $\gamma_k^{S_1\to S_2}$ | $\gamma_k^{T_1\to T_2}$ | $\gamma_k^{T_3\to T_4}$ | $\gamma_k^{T_7\to T_8}$ |
|---|---|---|---|---|---|---|---|---|---|---|---|---|
| $\nu_{64}$ | 7.40 | 1.51 | 3.75 | 3.78 | 7.69 | 2.26 | 3.90 | 3.94 | 27.84 | 8.82 | 5.82 | 15.41 |
| $\nu_{65}$ | 8.04 | 3.23 | 3.50 | 3.53 | 8.41 | 3.71 | 3.60 | 3.63 | | | | |
| $\nu_{68}$ | 43.41 | 39.59 | 9.30 | 9.37 | 43.44 | 44.71 | 14.89 | 15.03 | 8.85 | 18.30 | 24.97 | 5.19 |
| $\nu_{69}$ | 30.53 | 25.66 | 8.76 | 8.83 | 28.66 | 31.06 | 14.88 | 15.02 | | | | |
| $\nu_{72}$ | 48.99 | 38.90 | 18.80 | 18.95 | 28.53 | 59.52 | 45.57 | 46.00 | 61.77 | 64.40 | 46.55 | 100.68 |
| $\nu_{73}$ | 69.99 | 62.95 | 18.35 | 18.49 | 58.73 | 80.53 | 48.11 | 48.56 | | | | |
| $\nu_{74}$ | 8.62 | 5.94 | 9.68 | 9.76 | 29.67 | 31.23 | 35.96 | 36.30 | 14.07 | 38.64 | 16.13 | 7.17 |
| $\nu_{75}$ | 24.70 | 23.32 | 9.54 | 9.61 | 14.90 | 38.69 | 34.95 | 35.28 | | | | |
| $\nu_{76}$ | 139.88 | 118.74 | 50.97 | 51.38 | 140.75 | 134.49 | 24.22 | 24.45 | 39.39 | 59.60 | 89.75 | 22.67 |
| $\nu_{77}$ | 148.02 | 126.14 | 53.48 | 53.91 | 149.24 | 142.49 | 24.17 | 24.40 | | | | |

Table S7: Coupling parameters along all $e'$ mode pairs from $\nu_{64}$ to $\nu_{77}$ in meV.

Table S8: Coupling parameters along all $e''$ mode pairs in meV.

| | $\lambda_k^{T_1\to T_3}$ | $\lambda_k^{T_1\to T_4}$ | $\lambda_k^{T_1\to T_5}$ | $\lambda_k^{T_1\to T_6}$ | $\lambda_k^{T_2\to T_3}$ | $\lambda_k^{T_2\to T_4}$ | $\lambda_k^{T_2\to T_5}$ | $\lambda_k^{T_2\to T_6}$ |
|---|---|---|---|---|---|---|---|---|
| $\nu_1$ | 9.56 | 9.63 | 11.41 | 11.62 | 7.81 | 7.86 | 9.32 | 9.49 |
| $\nu_2$ | 11.32 | 11.39 | 13.50 | 13.75 | 12.85 | 12.94 | 15.35 | 15.63 |
| $\nu_5$ | 9.19 | 9.25 | 10.96 | 11.16 | 17.72 | 17.84 | 21.15 | 21.55 |
| $\nu_6$ | 17.84 | 17.97 | 21.28 | 21.68 | 12.66 | 12.75 | 15.11 | 15.39 |
| $\nu_{15}$ | 3.40 | 3.42 | 4.05 | 4.13 | 12.43 | 12.52 | 14.84 | 15.11 |
| $\nu_{16}$ | 13.17 | 13.27 | 15.72 | 16.01 | 1.40 | 1.41 | 1.67 | 1.71 |
| $\nu_{23}$ | 14.05 | 14.15 | 16.76 | 17.07 | 19.93 | 20.07 | 23.80 | 24.24 |
| $\nu_{24}$ | 20.09 | 20.23 | 23.97 | 24.41 | 13.87 | 13.97 | 16.56 | 16.87 |
| $\nu_{30}$ | 14.35 | 14.45 | 17.12 | 17.44 | 15.38 | 15.49 | 18.36 | 18.71 |
| $\nu_{31}$ | 15.43 | 15.54 | 18.40 | 18.74 | 14.59 | 14.69 | 17.41 | 17.74 |
| $\nu_{45}$ | 10.72 | 10.79 | 12.79 | 13.02 | 5.60 | 5.64 | 6.69 | 6.81 |
| $\nu_{46}$ | 4.50 | 4.53 | 5.36 | 5.46 | 11.54 | 11.63 | 13.78 | 14.04 |
| $\nu_{51}$ | 17.12 | 17.24 | 20.42 | 20.80 | 11.82 | 11.90 | 14.11 | 14.38 |
| $\nu_{52}$ | 13.10 | 13.19 | 15.63 | 15.92 | 19.36 | 19.50 | 23.11 | 23.54 |
| $\nu_{81}$ | 18.27 | 18.40 | 21.80 | 22.20 | 12.93 | 13.02 | 15.44 | 15.73 |
| $\nu_{82}$ | 12.32 | 12.41 | 14.70 | 14.97 | 18.39 | 18.52 | 21.96 | 22.36 |
| $\nu_{83}$ | 12.61 | 12.70 | 15.04 | 15.32 | 10.22 | 10.29 | 12.20 | 12.43 |
| $\nu_{84}$ | 11.34 | 11.42 | 13.53 | 13.78 | 14.25 | 14.35 | 17.02 | 17.33 |
| $\nu_{85}$ | 11.50 | 11.58 | 13.72 | 13.98 | 9.53 | 9.59 | 11.37 | 11.59 |
| $\nu_{86}$ | 7.27 | 7.32 | 8.67 | 8.83 | 10.46 | 10.54 | 12.49 | 12.73 |

| | $S_0$ Geometry | | | | | | | $S_1$ Geometry | | | | | | | $T_1$ Geometry | | | | | | |
|---|---|---|---|---|---|---|---|---|---|---|---|---|---|---|---|---|---|---|---|---|---|
| | $T_2$ | $T_3$ | $T_4$ | $T_5$ | $T_6$ | $T_7$ | $T_8$ | $T_2$ | $T_3$ | $T_4$ | $T_5$ | $T_6$ | $T_7$ | $T_8$ | $T_2$ | $T_3$ | $T_4$ | $T_5$ | $T_6$ | $T_7$ | $T_8$ |
| $S_1$ | - | 6.4 | 6.2 | 10.3 | - | - | - | - | 8.7 | 6.7 | 9.2 | - | - | - | - | 8.7 | 6.7 | 9.2 | - | - | - |
| $S_2$ | - | 6.0 | 6.2 | 10.5 | - | - | - | - | 3.5 | 6.5 | 11.5 | - | - | - | - | 3.5 | 6.5 | 11.5 | - | - | - |
| $T_1$ | 0.1 | 15.8 | 15.4 | 22.5 | - | 0.1 | - | 0.1 | 24.0 | 14.3 | 19.1 | - | 0.1 | - | 0.1 | 24.0 | 14.3 | 19.1 | - | 0.1 | - |
| $T_2$ | - | 15.1 | 15.3 | 22.9 | - | - | 0.1 | - | 9.5 | 16.2 | 26.0 | - | - | 0.1 | - | 9.5 | 16.2 | 26.0 | - | - | 0.1 |
| $T_3$ | - | - | 2.8 | - | 29.4 | 9.2 | 9.2 | - | - | 2.7 | - | 32.6 | 6.9 | 1.9 | - | - | 2.7 | - | 32.6 | 6.9 | 1.9 |
| $T_4$ | - | - | - | - | 29.3 | 9.3 | 9.5 | - | - | - | 0.6 | 25.0 | 9.0 | 19.0 | - | - | - | 0.6 | 25.0 | 9.0 | 19.0 |
| $T_5$ | - | - | - | - | 0.3 | 8.2 | 7.9 | - | - | - | - | 4.9 | 9.9 | 8.5 | - | - | - | - | 4.9 | 9.9 | 8.5 |

Table S9: All non-zero spin-orbit couplings in $cm^{-1}$ at the ground state geometry

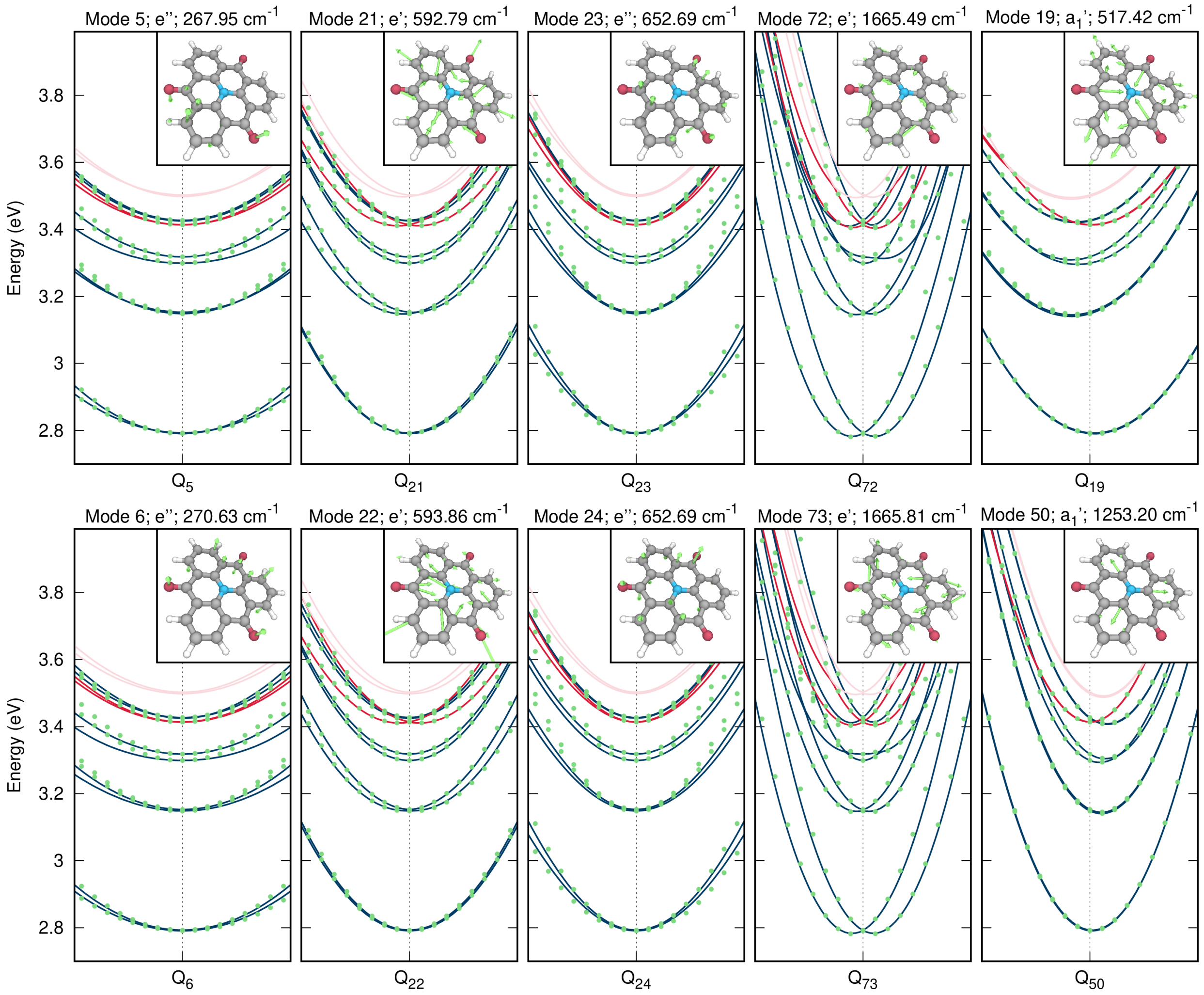


Figure S1: Energy of the Singlet (Red) and Triplet (Blue) states as the molecule is projected along all modes considered in the Dynamics. The green points represent the energy levels given by the LVC Hamiltonian along the normal modes. The inset image shows the mode on the molecule

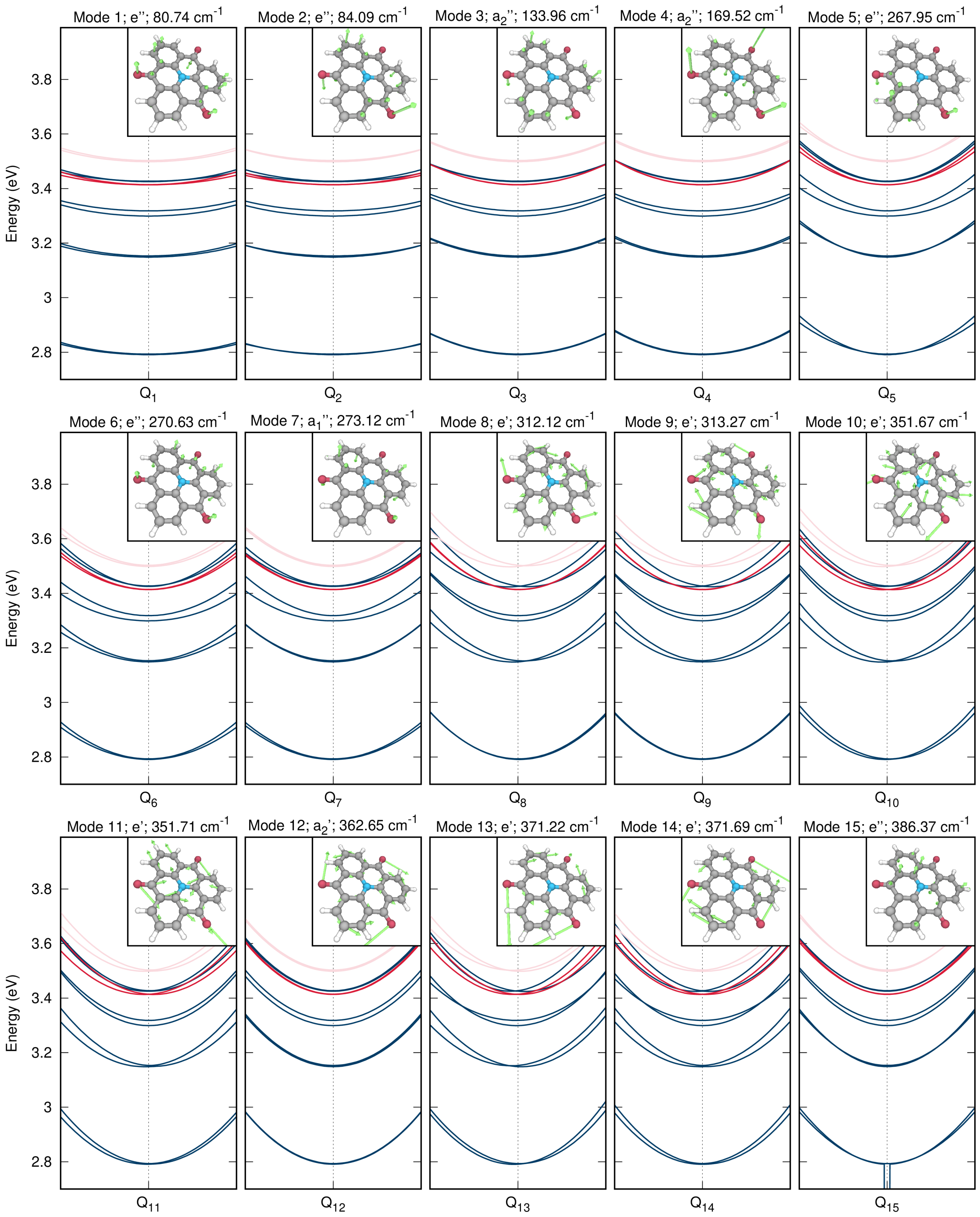


Figure S2: Energy of the Singlet (Red) and Triplet (Blue) states as the molecule is projected along modes 1 to 15. The inset image shows the mode on the molecule

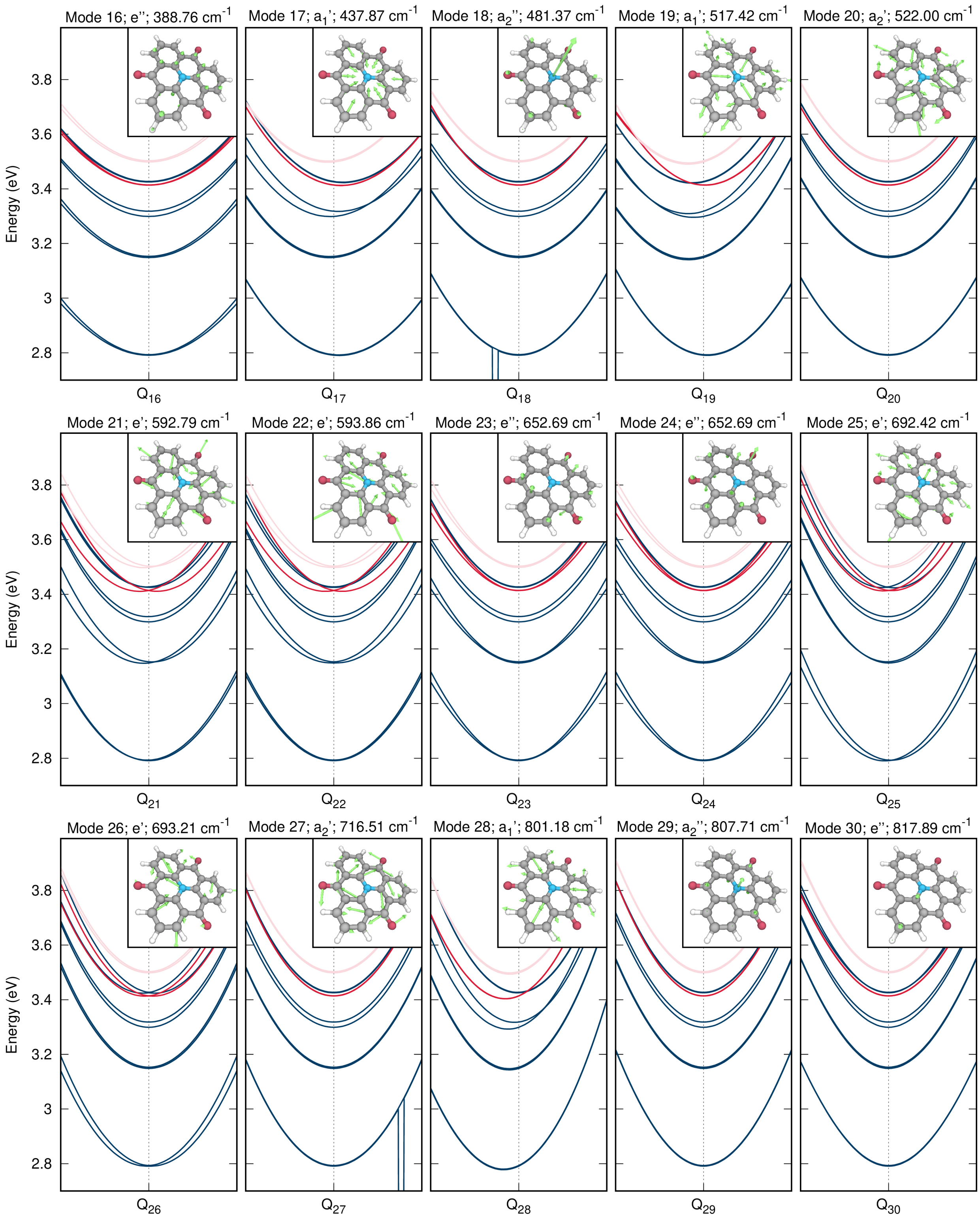


Figure S3: Energy of the Singlet (Red) and Triplet (Blue) states as the molecule is projected along modes 16 to 30. The inset image shows the mode on the molecule

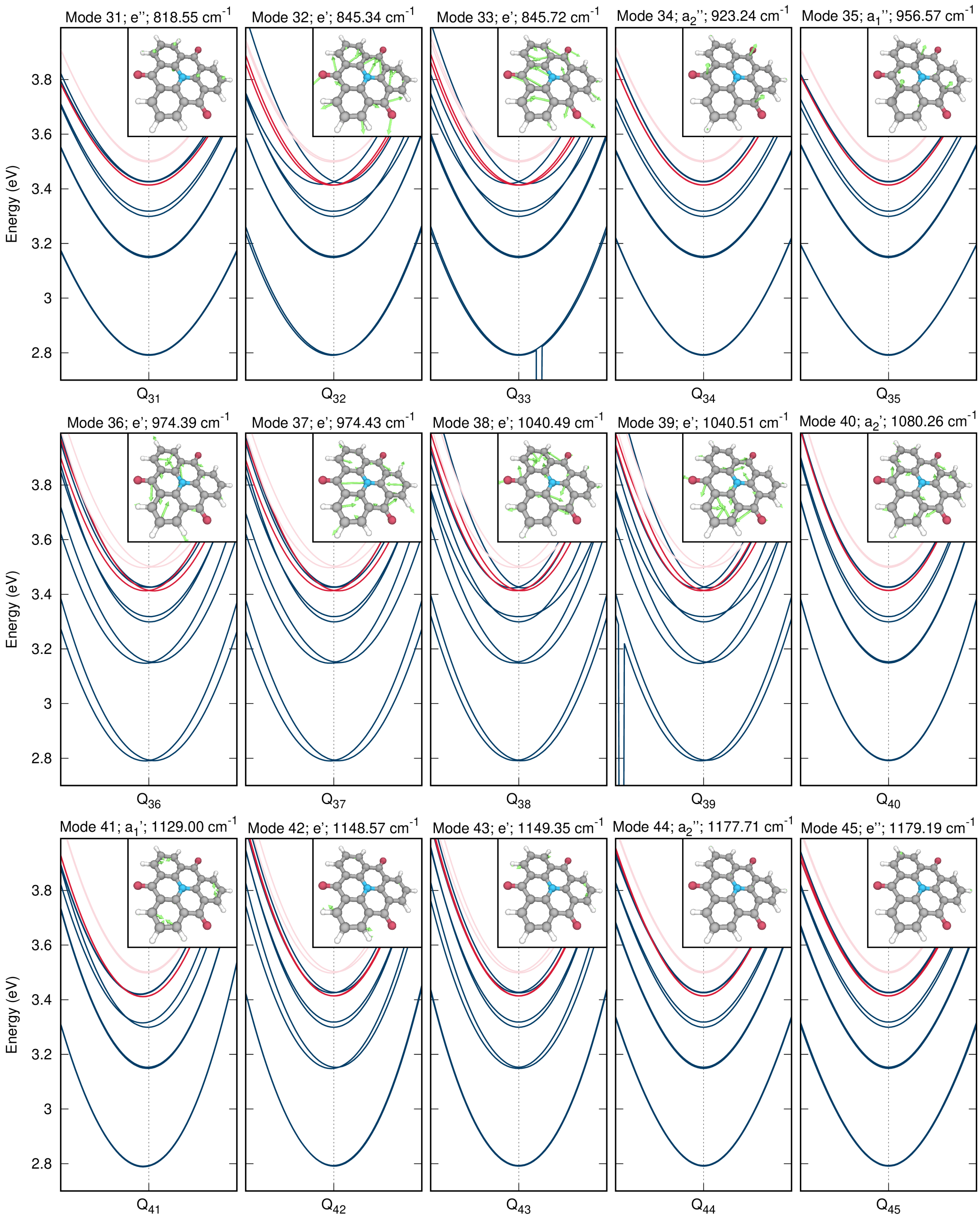


Figure S4: Energy of the Singlet (Red) and Triplet (Blue) states as the molecule is projected along modes 31 to 45. The inset image shows the mode on the molecule

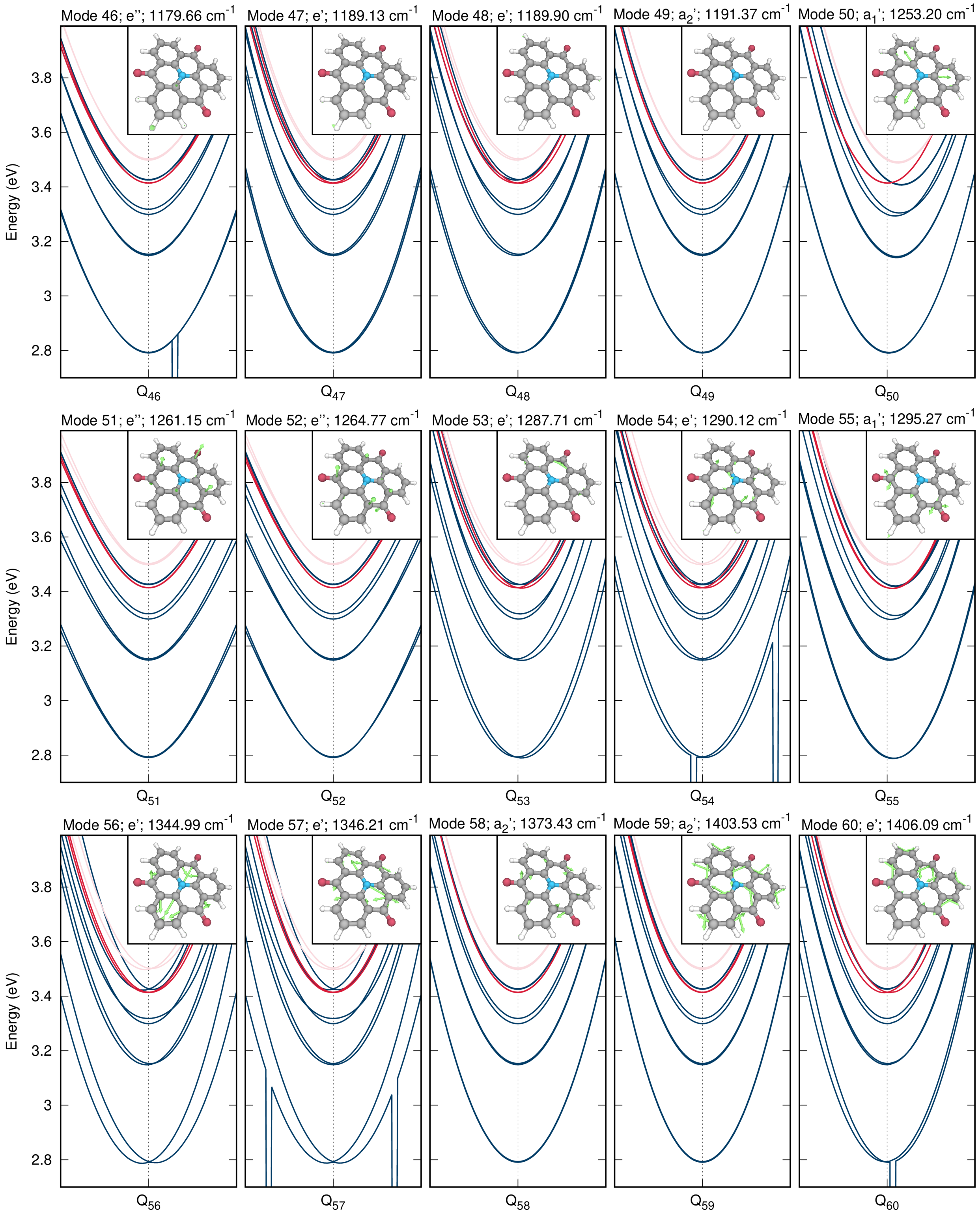


Figure S5: Energy of the Singlet (Red) and Triplet (Blue) states as the molecule is projected along modes 46 to 60. The inset image shows the mode on the molecule

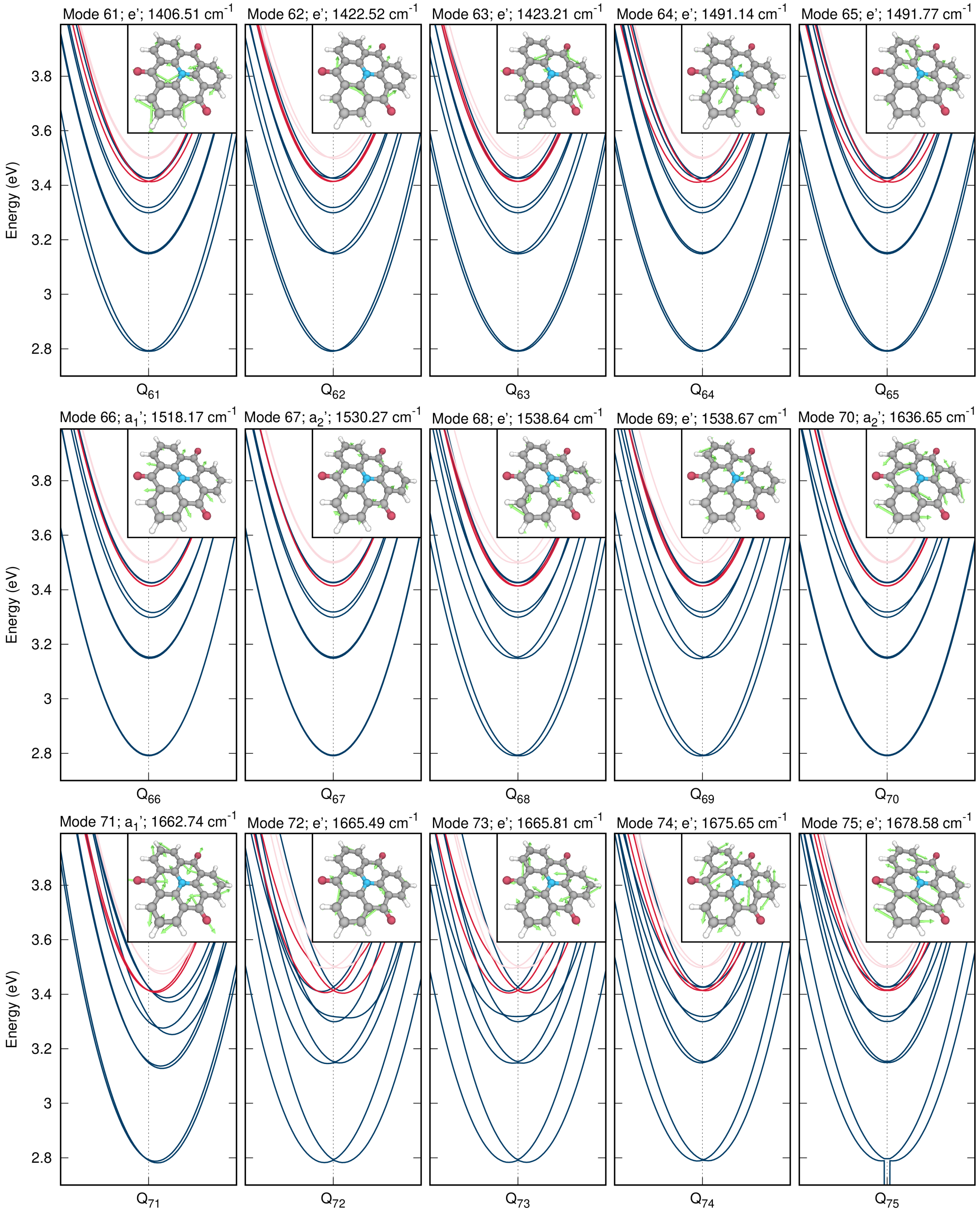


Figure S6: Energy of the Singlet (Red) and Triplet (Blue) states as the molecule is projected along modes 61 to 75. The inset image shows the mode on the molecule

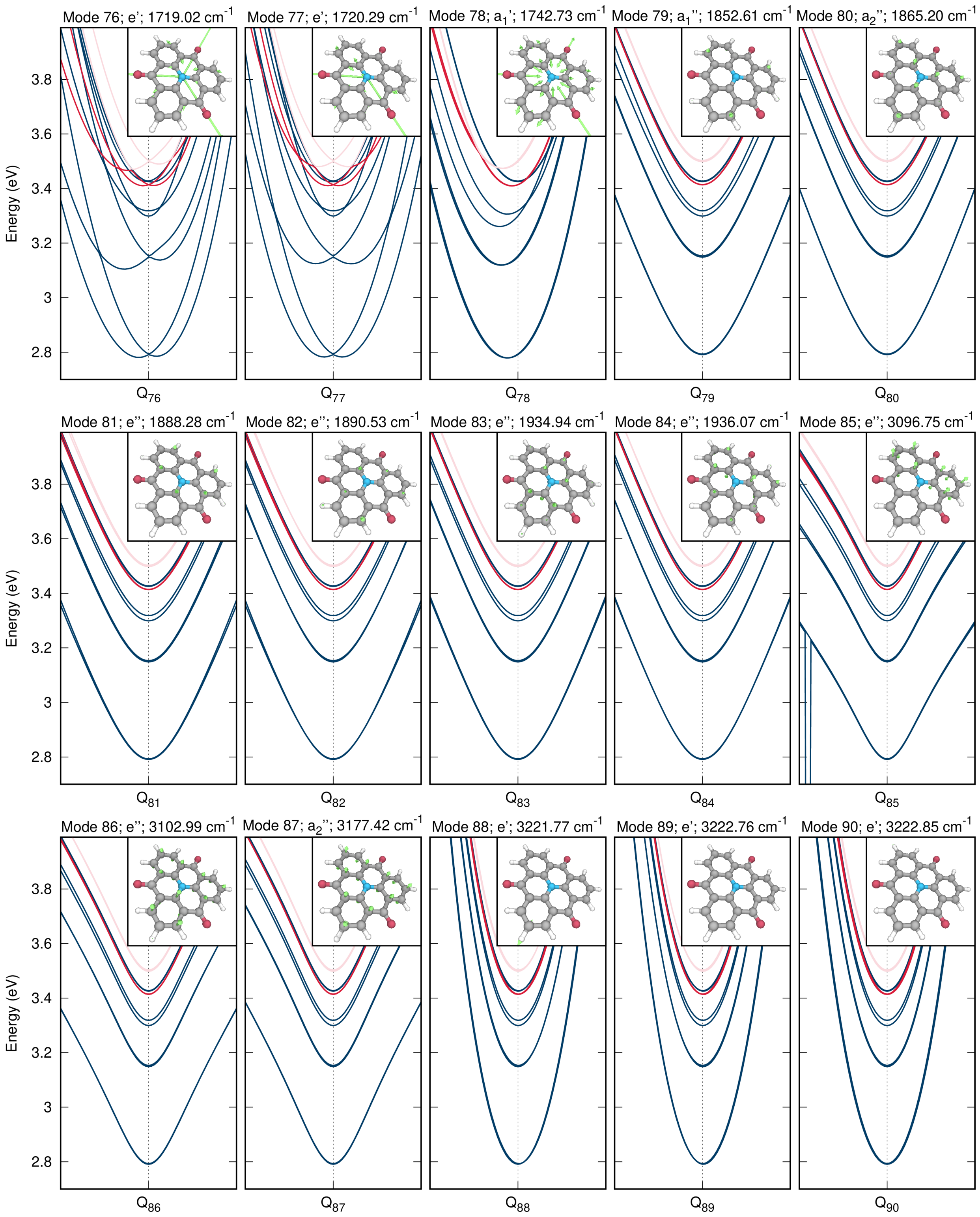


Figure S7: Energy of the Singlet (Red) and Triplet (Blue) states as the molecule is projected along modes 76 to 90. The inset image shows the mode on the molecule

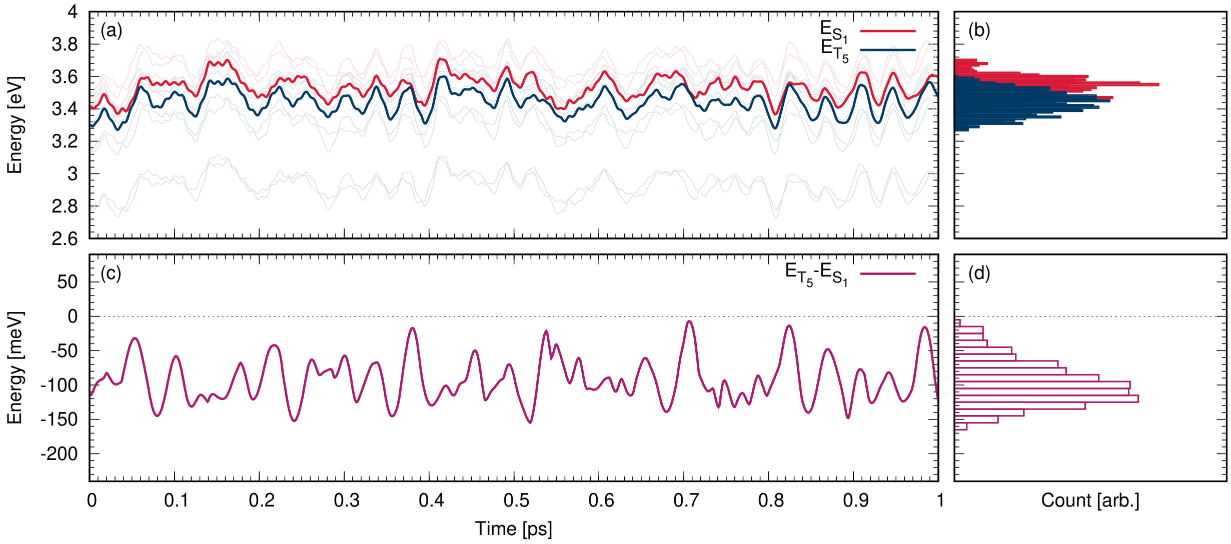


Figure S8: Ab-Initio Molecular Dynamics on a TANGO monomer with a time-step of 0.5fs. The temperature is maintained at 77K via a Berendsen thermostat with a time constant of 10fs. Throughout the simulation, the temperature of the system has a standard deviation of 7K arount 77K. (a) Singlet (red) and Triplet (blue) state energies as a function of time. Highlighted are the $S_1$ and $T_5$ energies. (b) Histogram of the $S_1$ (red) and $T_5$ (blue) energies throughout the MD run. (c) Difference between the $T_5$ and $S_1$ energies as a function of time. (d) Histogram of the difference between the $S_1$ and $T_5$ energies throughout the MD run. Here, the standard deviation is calculated to be 31 meV.

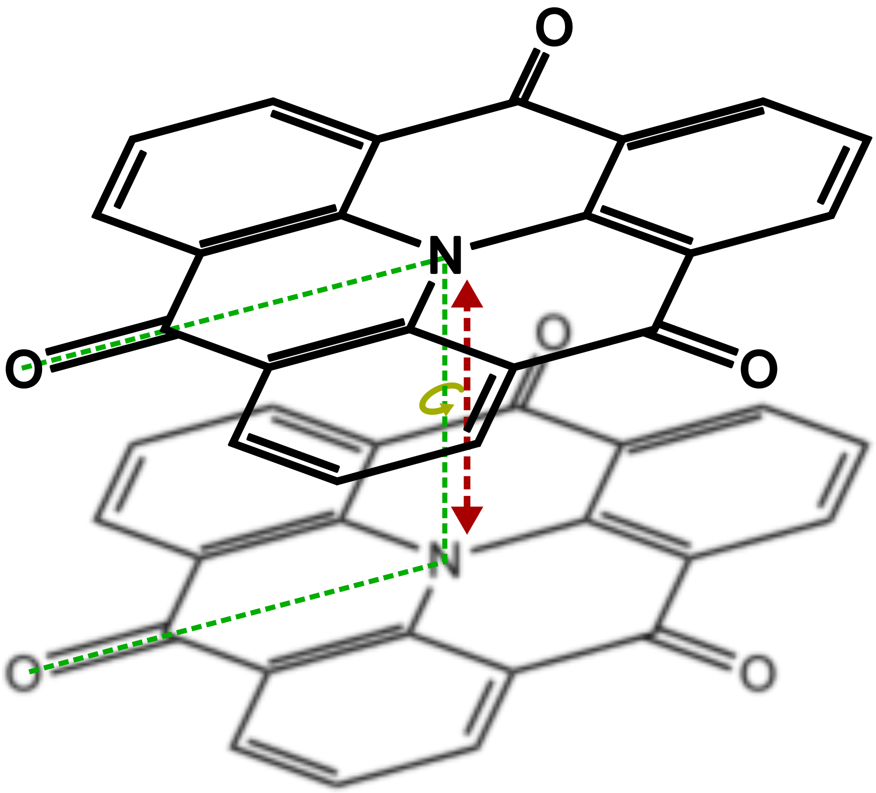


Figure S9: Schematic of the TANGO Dimer system, showing the dimer spacing (red) and dimer angle (green) parameters.
